# PPP1R26 drives hepatocellular carcinoma progression by controlling glycolysis and epithelial-mesenchymal transition

**DOI:** 10.1186/s13046-022-02302-8

**Published:** 2022-03-15

**Authors:** Yang Yang, Pengwei Ren, Xiaofeng Liu, Xiaoyan Sun, Chunfeng Zhang, Xiaojuan Du, Baocai Xing

**Affiliations:** 1 Hepatopancreatobiliary Surgery Department I, Key laboratory of Carcinogenesis and Translational Research (Ministry of Education/Beijing), Peking University Cancer Hospital & Institute, Beijing, 100142 China; 2 Department of Cell Biology, School of Basic Medical Sciences, Peking University Health Science Center, Beijing, 100083 China; 3 Department of Medical Genetics, School of Basic Medical Sciences, Peking University Health Science Center, Beijing, 100083 China

**Keywords:** PPP1R26, PKM2, glycolysis, epithelial-mesenchymal transition, hepatocellular carcinoma

## Abstract

**Background:**

Hepatocellular carcinoma (HCC) is usually diagnosed at an advanced stage due to rapid progression. Glycolysis supports anabolic growth and metastasis to promote HCC progression. However, the molecular mechanisms linking glycolysis and metastasis in HCC are not completely defined.

**Methods:**

The expression of PPP1R26 in human HCC tissues was evaluated by immunohistochemistry, and the clinical significance of PPP1R26 in the progression and prognosis of the HCC patients were analyzed. The PPP1R26-binding proteins were determined by mass spectrometry analysis. The function of PPP1R26 in glycolysis, EMT and tumorigenesis were evaluated in HCC cells. Glucose uptake and tumor growth were evaluated using PET imaging in mouse xenografts *in vivo*. Protein binding was confirmed by co-immunoprecipitation and immunofluorescence co-localization. Protein-RNA binding was determined by RNA-immunoprecipitation (RIP) experiment. The binding of protein on the promoter was evaluated by chromatin immunoprecipitation assay (ChIP).

**Results:**

PPP1R26 is upregulated in human HCC tissues and its upregulation is significantly associated with metastasis and the poor survival of the patients. PPP1R26 activates glycolysis in HCC cells and in mouse xenografts *in vivo*. PPP1R26 drives glycolysis by binding to PTBP1 to facilitate the mRNA splicing of *PKM2*. Simultaneously, overexpressed PPP1R26 induces the nuclear accumulation of PKM2 to inhibit the expression of E-cadherin further to drive EMT. Mechanistically, PPP1R26 binds with Ser37-phosphorylated PKM2 and TGIF2 in the nucleus and blocks the binding of TGIF2 with *CDH1* promoter to inhibit the transcription of *CDH1*.

**Conclusion:**

PPP1R26 promotes glycolysis by enhancing PKM2 splicing and simultaneously activates EMT by forming a PPP1R26-PKM2-TGIF2 complex to drive HCC progression. Therefore, targeting PPP1R26 attenuates HCC progression and provides a potential therapeutic strategy for the HCC patients with upregulation of PPP1R26.

**Supplementary Information:**

The online version contains supplementary material available at 10.1186/s13046-022-02302-8.

## Background

Hepatocellular carcinoma (HCC) is the third leading cause of tumor death worldwide, representing up to 75–85% of all primary liver malignancies, and causes a major global health problem [1, 2]. Generally, HCC is often diagnosed at an advanced stage with a high metastasis rate, resulting in the primary cause of mortality [2]. Thus, elucidation of the molecular mechanisms underlying HCC progression is expected to improve the prognosis for HCC patients.

Cancer cells acquire energy through aerobic glycolysis even under normoxic conditions, known as the Warburg effect [3]. Aerobic glycolysis was firstly found in rat liver carcinoma by Warburg and acts as a hallmark of liver cancer [3]. In addition to supplying ATP for cancer cell metabolism, the final product lactate and the metabolic intermediates of aerobic glycolysis provide a favorable microenvironment for tumor cell growth and metastasis [4, 5]. The Warburg effect has been featured by enhanced glucose uptake and lactate production [6]. Due to the importance of glycolysis in tumor progression, the ^18^F-fluorodeoxyglucose (FDG) positron emission tomography (PET) imaging has been applied to diagnose cancers and monitor tumor progression in the clinic by quantitating glucose uptake [7]. Besides driving cell proliferation, elevated glycolysis also promotes immune evasion, tumor invasion, metastasis, angiogenesis and drug resistance in HCC [3, 8–11]. Thus, exploration of the molecules regulating glycolysis may provide new insight into the understanding of HCC progression.

Pyruvate kinase (PK) is a rate-limiting enzyme in glycolysis and is responsible for catalyzing phosphoenolpyruvate (PEP) to pyruvate [12]. The expression of PK is tightly controlled to maintain cellular homeostasis. PK has four isoforms including PKL, PKR, PKM1 and PKM2. PKL and PKR are commonly expressed in kidney, liver and red blood cells [13]. PKM1 and PKM2 are encoded by *PKM* gene and generated by mutually exclusive alternative splicing of exons 9 and 10, respectively [12]. PKM2 is ubiquitously upregulated in multiple malignancies, whereas the expression of PKM1 is generally restricted in normal tissues [12, 14]. The replacement of PKM1 by PKM2 in cancer cells elevates the glycolysis rate to promote cell proliferation [15]. An increased ratio of *PKM2/PKM1* mRNA in cancer cells is controlled by three heterogeneous nuclear ribonucleoproteins (hnRNPs) including PTBP1 (also known as hnRNPI), hnRNPA1 and hnRNPA2 [16]. Emerging studies found that PTBP1 promotes tumorigenesis by functioning as a splicing suppressor [17]. As for *PKM* splicing, PTBP1 binds explicitly to the intronic fragments flanking exon 9 of the *PKM* pre-mRNA to inhibit splicing of *PKM1* and promote *PKM2* splicing [16, 18, 19]. Aside from the essential role in glycolysis, PKM2 also binds to TGIF2 (TGFB induced factor homeobox 2) in the nucleus to suppress the transcription of *CDH1* (cadherin 1), promoting the epithelial-mesenchymal transition (EMT) [20].

PPP1R26 (Protein Phosphatase1 Regulatory subunit 26), also known as KIAA0649 and NRBE3 (a Novel Retinoblastoma E3 ligase), was identified as a potential oncoprotein as it transforms NIH3T3 fibroblast cell and acts as a ubiquitin E3 ligase for RB to promote RB degradation through the proteasome-ubiquitination pathway [21, 22]. Although PPP1R26 inhibits the phosphatase activity of protein phosphatase 1 (PP1) complexes [23, 24], the downstream targets of PPP1R26 are still unclear. We previously found that PPP1R26 promotes breast cancer metastasis [22, 25]. However, the potential role of PPP1R26 in HCC and the underlying molecular mechanisms remain unknown. In this study, we evaluated the expression of PPP1R26 in human HCC tissues and determined the oncogenic role of PPP1R26 in HCC progression. Bioinformatics analysis indicates that PPP1R26 is positively related to glucose metabolism. We, therefore, set out to explore how PPP1R26 controls glycolysis and HCC progression, especially metastasis. We demonstrated that PPP1R26 promotes glycolysis by binding to PTBP1 to facilitate *PKM2* splicing. Meanwhile, overexpressed PPP1R26 induces the nuclear accumulation of PKM2 and forms a complex with p-Ser37-PKM2 and TGIF2 to inhibit the transcription of *CDH1*, thus down-regulating E-cadherin expression and accelerating epithelial-mesenchymal transition (EMT). Our findings demonstrate that glycolysis and EMT are simultaneously activated by upregulation of PPP1R26 to drive tumor progression in HCC, and provide PPP1R26 as a potential therapeutic target in HCC.

## Materials and methods

### Human tissue samples

Patients who underwent curative hepatectomy as initial treatment for HCC at Peking University Cancer Hospital between 2009 and 2011 were considered for enrollment. Patients with other malignancies, absence of paraffin-embedded clinical tissue specimens and with incomplete clinicopathological information were excluded. In total, 128 cases were eligible for analysis, including 128 paired tumor tissue and non-tumorous samples. Tumor staging was performed using the Barcelona Clinical Liver Cancer (BCLC) staging system [26]. All patients underwent follow-up evaluations until June 2016. This investigation was approved by the Ethics Committee of Peking University Cancer Hospital. Informed consent was obtained from each patient at the time of sample collection.

### Cell culture and reagents

SMMC7721, Huh7 and HepG2 cells were used in this study. HepG2 and SMMC7721 cells were cultured in DMEM (Gibco, Thermo Fisher Scientific, Waltham, MA, USA), and Huh7 cells were cultured in RPMI 1640 (Gibco, Thermo Fisher Scientific, Waltham, MA, USA). All media was supplemented with 10% fetal bovine serum (FBS). Cells were incubated in a humidified chamber of 5% CO_2_ at 37 °C. The reagents for this study are listed in Supplementary Table 1.

### Cell transfection

Cells were transfected with plasmid DNA or siRNA using Lipofectamine 2000 (Invitrogen, Carlsbad, CA, USA) according to the instructions provided by the manufacturer. Plasmid DNA was kept consistent with empty vector in transient transfection experiments. To construct stable PPP1R26 knockdown cell lines, pLKO.1-PPP1R26 shRNA or pLKO.1-ctrl shRNA were transfected into HEK293T cells with the packaging vectors pMD2.G and psPAX2 to produce lenti-viral particles. HCC cells were infected with lentiviruses delivering PPP1R26 shRNA or control shRNA to obtain stable PPP1R26 knockdown cell lines and control cell lines. Small interfering RNAs (siRNAs) targeting PPP1R26, PTBP1 or control siRNA were synthesized (GenePharma, Shanghai, China). The sequences of all shRNAs and siRNAs are listed in Supplementary Table 2.

### Cell proliferation assay

MTS assay and colony formation were used to determine cell proliferation. For the MTS assay, the CellTiter 96 AQueous One Solution Cell Proliferation Assay system (Promega, Madison, WA, USA) was used according to the manufacturer’s instructions. Briefly, HCC cells were seeded at a density of 1 × 10^3^ cells/well in 96-well plate. At different time points, 10 μl of MTS were added to each well in the plate and incubated for 4 h at 37 °C. Absorbance at 490 nm was measured using a Multiskan FC microplate photometer (Thermo Scientific, Waltham, MA, USA). For colony formation, HCC cells were seeded into 6-well plates at a density of 500 cells/well and cultured under normal conditions for about 14 days. Cell clones were fixed with 4% paraformaldehyde and stained with crystal violet. Visible colonies were counted under the microscope.

### Cell wound healing and transwell assays

The wound-healing experiment was performed as previously described [27]. The wound area was measured by ImageJ software. Transwell invasion and migration assays were performed using chambers (Corning, NY, USA) with or without Matrigel coating, respectively. Chambers were placed in 24-well plates. Cells were serum-starved overnight and plated to the upper compartments containing serum-free DMEM. DMEM containing 10% FBS was added to the lower compartments. After a 24 h incubation period at 37 °C, the migratory or invasive cells were stained with crystal violet and counted in five random fields.

### Glucose uptake and lactate production assays

After transfections, the culture medium was collected for measurement of glucose and lactate concentration while cells were harvested for preparation of cell lysates. Glucose concentration was measured by the Amplex-Red Glucose assay kit (Invitrogen, Carlsbad, CA, USA). Glucose uptake was calculated by deducting the glucose concentration in the media from the initial glucose concentration. For evaluating lactate production, cells were cultured under DMEM without pyruvate for 24 h after transfection, and the concentration of lactate was measured using the L-Lactate Assay Kit (Abcam, Cambridge, UK) according to the manufacturer’s instruction. All values were normalized based on the cell number.

### Extracellular acidification rate (ECAR)

The glycolysis stress test was performed using the Seahorse XF Glycolysis Stress Test Kit (Agilent Technologies, Palo Alto, CA, USA) according to the instructions provided by the manufacturer. The ECAR reported by this method reflected the main parameters of glycolysis, including glycolysis rate and glycolysis capacity. The ECAR value was normalized by the cell number per well on the website of Agilent (https://seahorseanalytics.agilent.com). The reagents for ECAR are listed in Supplementary Table 1.

### Immunoprecipitation assay

Immunoprecipitation was performed as previously described [28]. Briefly, cells were harvested and cell lysates were prepared in Buffer A (25 mM Tris-Cl pH 7.5, 150 mM KCl, 1 mM dithioerythritol, 2 mM ethylenediaminetetraacetic acid, 0.5 mM phenylmethylsulfonyl fluoride, and 0.2% Nonidet P-40) and used directly for immunoprecipitation. Antibody (1 μg) was incubated with 20 μL of Protein A Sepharose beads (GE Healthcare, Madison, WI, USA) in Buffer IPP500 (500 mM NaCl, 10 mM Tris-Cl pH 8.0, 0.2% Nonidet P-40) for 2 h at 4 °C. After centrifugation, the Ab-coupled beads were incubated with cell lysates for 2 h at 4 °C. After washing three times with Buffer B (10 mM Tris-Cl pH 8.0, 150 mM KCl, 5 mM MgCl_2_, 0.05% NP-40), the precipitates were subjected to Western blot using indicated antibodies.

### RNA immunoprecipitation (RIP)

RIP assay was carried out as previously described [28, 29]. In brief, 1 × 10^7^ cells were treated with UV irradiation and lysed in RIP lysis buffer. Then, cell extracts were immunoprecipitated with anti-PPP1R26-coated magnetic beads overnight at 4 °C. After being washed six times, the PPP1R26-immunoprecipitated complexes were incubated with proteinase K digestion buffer. RNA was extracted from the precipitates using phenol: chloroform: isoamyl alcohol (125:24:1) RNA extraction methods. The relative expression of RNA was determined by RT-qPCR and normalized to the input. Normal mouse IgG was used as a negative control.

### Chromatin Immunoprecipitation (ChIP)

ChIP was performed as described previously [30]. Briefly, 1% formaldehyde was added to the cells to cross-link nuclear proteins with genomic DNA, and 0.125 M glycine was added to stop the cross-linking. The cells were collected, centrifugated and resuspended in FA lysis buffer (1% SDS, 10 mM EDTA, protease inhibitors and 50 mM Tris-HCl pH 8.0) and genomic DNA was sonicated to get a length of approximately 300 to 1000 bp. Cell lysates were clarified by centrifugation, diluted by 1:10 in ChIP dilution buffer (0.01% SDS, 1.0% Triton X-100, 1.2 mM EDTA, 16.7 mM NaCl, protease inhibitors and 16.7 mM Tris-HCl pH 8.0) and incubated with anti-TGIF2 or control IgG overnight at 4 °C with rotation. ChIP-Grade Protein G Magnetic Beads (Cell Signaling Technology) were added to each reaction and incubated for 2 h. Protein G magnetic beads were pelleted by centrifugation and washed with the following buffers: low salt wash buffer (0.1% SDS, 1% Triton X-100, 2 mM EDTA, 150 mM NaCl and 20 mM Tris-HCl pH 8.0), high salt wash buffer (0.1% SDS, 1% Triton X-100, 2 mM EDTA, 500 mM NaCl and 20 mM Tris-HCl pH 8.0) and LiCl wash buffer (0.25 mM LiCl, 1% NP-40, 1% sodium deoxycholate, 1 mM EDTA and 10 mM Tris-HCl pH 8.0). Finally, beads were washed twice with 1 ml of TE buffer (1 mM EDTA and 10 mM Tris-HCl pH 8.0). The protein-DNA cross-linking was reversed by incubation with 200 mM NaCl and proteinase K for 2 h at 65 °C. DNA was extracted and subjected to real-time qPCR reaction.

### Cellular fractionation

Cellular fractionation was prepared as described previously [30]. Briefly, HCC cells were collected and resuspended gently in Buffer A (1 mM HEPES-KOH pH 7.9, 1.5 mM MgCl_2_, 10 mM KCl, 0.5 mM DTT, 0.5% NP-40), incubated on ice for 10 min and then centrifugated to collect supernatant as cytoplasmic fraction. The pellet were washed with Buffer A and suspended in Buffer T (1% NP-40, 450 mM NaCl, 50 mM Tris-Cl pH 7.4, 1 mM PMSF, 0.2 mM Na_3_VO_4_, 5 mM β-glycerophase, 20% glycerol, 2 mM DTT). The supernatant was collected as nuclear extracts after centrifugation at 12000 rpm for 30 min.

### Protein extraction from human HCC frozen tissues

Proteins for Western blot were extracted from human HCC frozen tissue samples using cold lysis buffer (1% NP-40, 50 mM Tris pH 7.4, 150 mM NaCl, 150 mM EDTA, 10% SDS, 10% Sodium deoxycholate, 1 × cocktail, 1 mM Na_3_VO_4_, 1 mM NaF, 1 mM PMSF). Protein concentration was measured by Coomassie brilliant blue G250 assay (Beyotime Biotechnology, Shanghai, China).

### Western blotting and antibodies

Western blot was performed as described previously [22]. Primary antibodies specific to PKM1 (1:1000), PKM2 (1:1000), PTBP1 (1:1000), phospho-Ser37 PKM2 (1:1000), E-cadherin (1:2000), Vimentin (1:1000), TOP1 (1:2000), Tubulin (1:3000), Flag (1:3000), GFP (1:5000) and β-actin (1:10000) were used. Anti-PPP1R26 was generated in our laboratory (1:1000) [13, 16]. The detailed information on antibodies for this study is listed in Supplementary Table 3.

### Real-time qPCR

Real-time qPCR was done as previously described [28]. Briefly, total RNA was extracted from cells by TRIzol Reagent (Invitrogen, Carlsbad, CA, USA) and reversely transcribed into cDNA. Reverse transcription was performed using PrimeScript™ RT Master Mix (Roche, Nutley, NJ, USA). Real-time PCR was performed with SYBR^®^ Premix Ex Taq™ GC (Roche, Nutley, NJ, USA) to determine the relative mRNA expression levels. The human β-actin was included as an internal control. Each sample was assessed in triplicate and each real-time qPCR experiment was independently repeated at least three times. Gene expression was presented as the ratio of target gene mRNA levels to the β-actin mRNA level. The primers were described in Supplementary Table 4.

### Immunohistochemistry (IHC)

IHC was performed as previously described [28]. The tissues were fixed in formalin and embedded in paraffin. Four-micrometer-thick sections of paraffin-embedded tissues were mounted on poly-L-lysine-coated slides. Then, the slides were deparaffinized in xylene and rehydrated with a gradient of ethanol and distilled water. Endogenous peroxidase activity was quenched with 3% hydrogen peroxide for 10 min at room temperature. After antigen retrieval, the sections were incubated with primary antibody overnight at 4 °C. The primary antibody was probed with a two-step Poly-HRP Anti-Mouse/Rabbit IgG Detection System (Zsbio, Beijing, China). The expression of proteins was classified according to a semi-quantitative score. The percentage of positive tumor cells were scored as following: 0% (0), 1–25% (1), 26–50% (2), 51–75% (3), and > 75% (4). The intensity was assessed as follows: negative staining (0), weak staining (1), moderate staining (2), and strong staining (3). The scores of staining intensity and proportion were multiplied for comprehensive evaluation. The expression of proteins in HCC tissues was scored independently by two senior pathologists.

### Immunofluorescence staining

Cells were fixed with 4% paraformaldehyde for 20 min, permeabilized with 0.1% Triton X-100 for 20 min at room temperature. Cells were incubated with primary antibodies overnight at 4 °C after blocking with 10% goat serum. After washing with 1 × PBS, the cells were incubated with secondary antibodies conjugated with DyLight 488 or DyLight 594 (1:100) (Earthox, Millbrae, CA, USA) for 1 h at 37 °C. The cells were stained with DAPI (Beyotime, Nantong, China) to visualize the nuclei.

### Xenograft mouse model

Female BALB/C nude mice aged 5-week (Beijing Vital River Laboratory Animal Technology Co., Ltd.) were randomly separated into three groups (*n* = 6, per group). SMMC7721-control shRNA, SMMC7721-PPP1R26 shRNA, Huh7-control-shRNA, Huh7-PPP1R26-shRNA, Huh7-PPP1R26-shRNA1 + GFP-PTBP1 or Huh7-PPP1R26-shRNA2 + GFP-PTBP1 cells (3 × 10^6^) were injected subcutaneously into the right and left flanks in each mouse, respectively. The tumor volume was measured every four days and calculated using the formula length×width^2^ × 0.5 (mm^3^). The tumor size, volume and weight were measured when the mice were sacrificed at day 28 (SMMC7721) or day 35 (Huh7) post-implantation. The expression of PPP1R26 and PKM2 was evaluated by Western blot and immunohistochemistry. Animal operations were performed according to the National Institutes of Health guidelines. Studies on animals obtained the approval of the Institutional Animal Care and Use Committee of Peking University Health Science Center (Ethics Approval License: LA2020428).

### MICRO-PET/CT imaging

SMMC7721/Huh7-Ctrl shRNA or SMMC7721/Huh7-PPP1R26 shRNA cells were subcutaneously injected into the nude mice. When tumor diameter reached up to 1 cm, mice were analyzed on a Super Nova PET/CT scanner (PINGSENG Healthcare [KunShan] Inc., Jiangsu, China). After mice had fasted overnight before the PET scans, the xenograft-bearing mice were injected with 18.5 MBq (0.5 mCi, 200 μL) of ^18^F-FDG via the tail vein. Scanning began 45 min after injection. The images were reconstructed using a three-dimensional ordered subsets expectation maximum algorithm (OSEM) without attenuation correction. PET images were acquired for 10 min and reconstructed with attenuation correction based on the CT data (CT-AC reconstruction).

### Single-sample gene set enrichment analysis (ssGSEA)

Gene signatures of metabolism were downloaded from Reactome Pathway Database (https://reactome.org/). The identifiers of glucose metabolism, fatty acid metabolism and amino acid metabolism are R-HSA-70326, R-HSA-8978868 and R-HSA-71291, respectively. Gene expression profiles of HCC patients in the TCGA liver hepatocellular carcinoma adenocarcinoma (LIHC) dataset (*n* = 374) was downloaded from the TCGA Data Portal (https://portal.gdc.cancer.gov/). Based on the expression of metabolic genes, the ssGSEA algorithm of R package “GSVA” was applied to calculate the metabolic score of each sample in TCGA LIHC dataset. Metabolic level of each sample was reflected by ssGSEA scores.

### Statistical analyses

Statistical analysis was performed using SPSS software version 22.0 (IBM, Armonk, NY, USA). The association between the expression of PPP1R26 and clinicopathological parameters in the HCC cohort was assessed by the chi-square test. Overall survival and its association with PPP1R26 protein expression were evaluated using the Kaplan-Meier method followed by the log-rank test. Subsequent univariate and multivariate analysis of prognostic factors was conducted using the Cox regression model. Data are presented as mean ± SEM or mean ± SD. Two-tailed Student’s t-tests were used to assess the differences between the two groups. One-way analysis of variance (ANOVA) with post hoc analysis LSD test was used to analyze differences among more than three groups. Kruskal-Wallis test was performed for nonparametric comparison. *P* < 0.05 was considered to indicate statistical significance. *P < 0.05, **P < 0.01, ***P < 0.001 and ****P < 0.0001 for all analyses.

## Result

### PPP1R26 is upregulated in HCC and is an independent diagnostic factor predicting worse prognosis of the patients

**Fig. 1:**
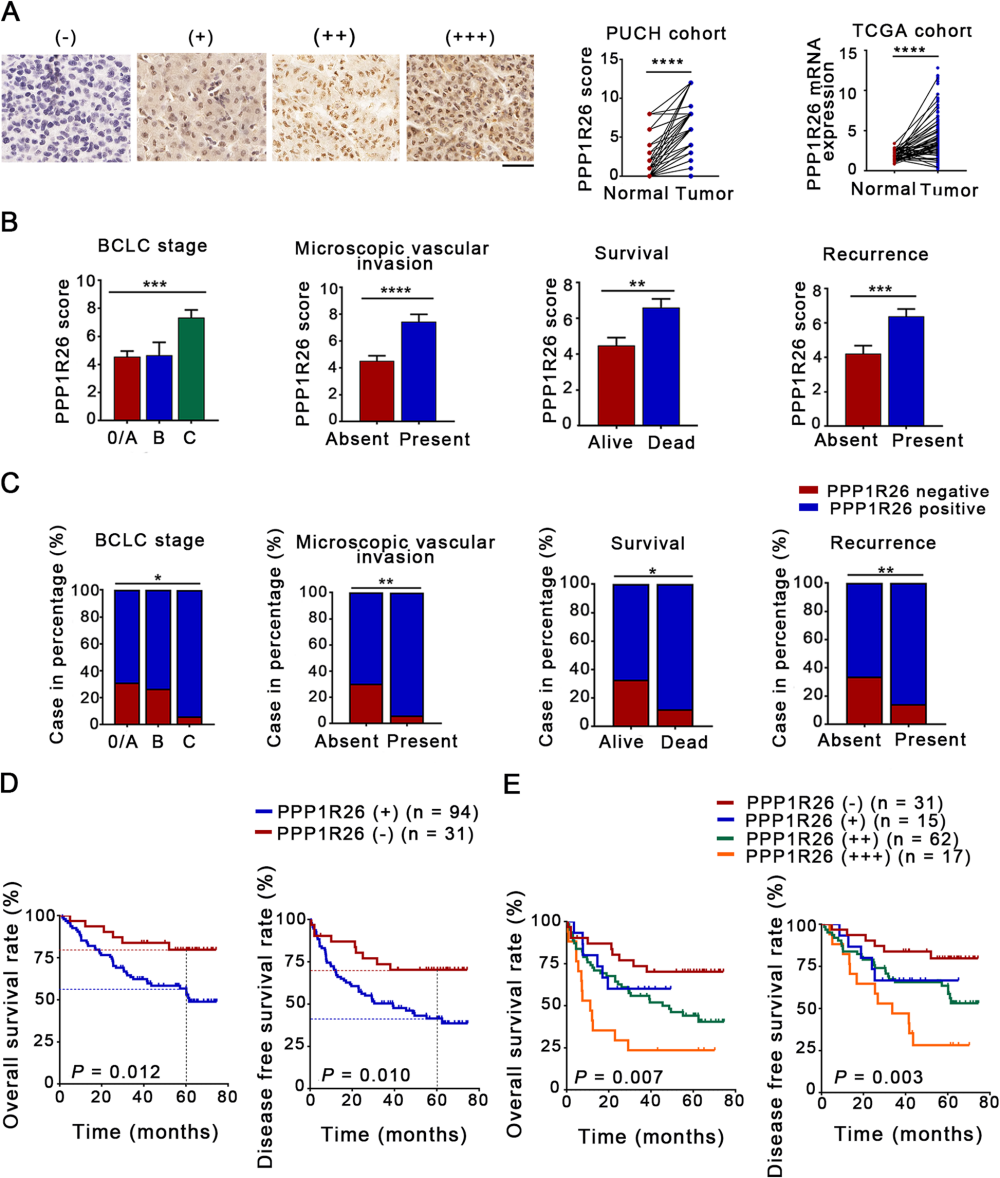
PPP1R26 is upregulated in HCC tumor tissues and related to a worse prognosis. **A** Staining intensity of PPP1R26 in HCC tissues is classified as negative staining (−, score 0), weak staining (+, score 1–4), moderate staining (++, score 5–8), and strong staining (+++, score 9–12) (left); IHC score of PPP1R26 in normal and HCC tissues in PUCH cohort (middle); PPP1R26 mRNA expression in TCGA HCC tissues (right). Scale bars represent 50 μm. **B** Staining scores of PPP1R26 in HCC tissues for each BCLC stage, the negative or positive of microscopic vascular invasion, survival status and the absence or presence of recurrence. **C** The percentage of the patients with PPP1R26 negative and positive expression in each group according to BCLC stages, survival, recurrence, and microscopic vascular invasion. **D** Kaplan-Meier survival curves of overall survival (OS) and disease-free survival (DFS) time in patients with positive or negative PPP1R26 expression. **E** Kaplan-Meier analysis of OS and DFS in 125 HCC patients stratified by PPP1R26 (−), PPP1R26 (+), PPP1R26 (++) and PPP1R26 expression (+++). Data information: In (**B-C**), data are presented as mean ± SEM. Statistical significance was assessed using two-tailed t-tests (**A, B & C**) or one-way ANOVA with post hoc analysis LSD test (**B & C**). In (**D-E**), *P* values were determined by log-rank test. *P < 0.05, **P < 0.01, ***P < 0.001 and ****P < 0.0001

To evaluate the expression level of PPP1R26 in human HCC tissues, IHC was performed. PPP1R26 is localized in the nucleus in tumor cells (Fig. 1A left) and upregulated in the HCC tumor tissues compared with their non-tumorous counterparts (Fig. 1A middle). Analysis of a 424-case cohort from The Cancer Genome Atlas (TCGA) database also showed that the expression of PPP1R26 is significantly upregulated in tumor tissues compared with adjacent normal tissues (Fig. 1A right). Additionally, the association of PPP1R26 with clinical characteristics was analyzed by chi-square test. The upregulation of PPP1R26 was markedly correlated with clinicopathological features including higher BCLC stages, worse survival, time to recurrence and microscopic vascular invasion (Fig. 1B). The ratio of the patients with positive PPP1R26 expression was also correlated with these clinicopathological characteristics (Table 1, Fig. 1C).

**Table 1:** Clinicopathological characteristics of HCC patients according to PPP1R26 expression

Variables	PPP1R26-negative (***n*** = 31)	PPP1R26-positive (***n*** = 97)	***P***-value
**Sex**			
Male	28	83	0.497
Female	3	14	
**Age (years)**			
≤ 60	23	63	0.34
>60	8	34	
**Tumor size (cm)**			
≤ 5	19	50	0.343
>5	12	47	
**Tumor number**			
Single	27	77	0.338
Multiple	4	20	
**Microscopic vascular invasion**			
Absent	29	66	0.005
Present	2	31	
**AFP (ng/ml)** ^a^			
≤ 200	22	62	0.375
>200	8	34	
**Cirrhosis**^a^			
Negative	18	65	0.327
Positive	13	31	
**HBV**^a^			
Negative	5	21	0.67
Positive	26	75	
**HCV**			
Negative	30	92	0.658
Positive	1	5	
**Edmondson-Steiner grade**			
1,2	24	77	0.816
3,4	7	20	
**BCLC stage**			
0 or A	25	55	0.017
B	4	11	
C	2	31	

^a^Data for AFP, cirrhosis, HBV were missing for two, one and one patients, respectively

To determine if PPP1R26 expression affects the prognosis of HCC patients, we analyzed the association of PPP1R26 expression with the survival of HCC patients. The 5-year overall survival (OS) in the PPP1R26-positive group (53.1%) was substantially lower than those in the PPP1R26-negative group (79.7%) (*P* < 0.05) (Fig. 1D left). The 5-year disease-free survival (DFS) in the PPP1R26-positive group (41.4%) was also significantly lower than that in the PPP1R26-negative group (70.3%) (Fig. 1D right). Moreover, when stratified by semi-quantitative scores of IHC, the OS and DFS of the HCC patients decreased along with the increased expression level of PPP1R26 (Fig. 1E), indicating that PPP1R26 is a potential prognosis marker in HCC.

We further evaluated the prognostic role of PPP1R26 expression in HCC patients. Univariate Cox regression analysis revealed that the PPP1R26 expression level, tumor number, microscopic vascular invasion, Edmondson-Steiner grade, BCLC stage and AFP level were the significant prognostic indicators in HCC (*P* < 0.05, respectively) (Table 2). In the multivariate model, PPP1R26 expression was related to poorer survival (HR = 2.893, 95% CI, 1.227–6.821, *P* = 0.015) (Table 3), indicating that high expression level of PPP1R26 was an independent prognostic factor in HCC.

**Table 2:** Univariate Cox regression analysis of potential poor prognostic factors for HCC patients in our study

Variables	Hazard ratio (95% confidence interval)	***P***-value
**PPP1R26**		
Negative	1	
Positive	2.894 (1.231–6.805)	0.015
**Sex**		
Female	1	
Male	0.781 (0.332–1.836)	0.570
**Age (years)**		
≤ 60	1	
>60	0.953 (0.519–1.75)	0.877
**Tumor size (cm)**		
≤ 5	1	
>5	0.953 (0.519–1.75)	0.877
**Tumor number**		
Single	1	
Multiple	0.369 (0.206–0.662)	0.001
**Microscopic vascular invasion**		
Negative	1	
Positive	2.093 (1.124–3.898)	0.020
**Cirrhosis**		
Negative	1	
Positive	0.799 (0.11–5.794)	0.824
**HBV**		
Negative	1	
Positive	1.315 (0.723–2.39)	0.370
**HCV**		
Negative	1	
Positive	0.704 (0.373–1.328)	0.278
**Edmondson-Steiner grade**		
1,2	1	
3,4	3.721 (2.1–6.595)	0.000
**BCLC stage**		
0 or A	1	
B	2.794 (1.278–6.107)	0.010
C	2.812 (1.51–5.236)	0.001
**AFP (ng/ml)**		
≤ 200	1	
>200	2.418 (1.374–4.254)	0.002

**Table 3:** Multivariate Cox regression analysis of potential poor prognostic factors for HCC patients in our study

Variables	Hazard ratio (95% confidence interval)	***P***-value
**PPP1R26**		
Negative	1	
Positive	2.893 (1.227–6.821)	0.015
**Tumor number**		
Single	1	
Multiple	2.494 (1.381–4.504)	0.002
**Edmondson-Steiner grade**		
1,2	1	
3,4	3.777 (2.121–6.728)	0.000
**AFP (ng/ml)**		
≤ 200	1	
>200	2.097 (1.188–3.704)	0.011

### PPP1R26 promotes HCC cell proliferation, migration and invasion

**Fig. 2:**
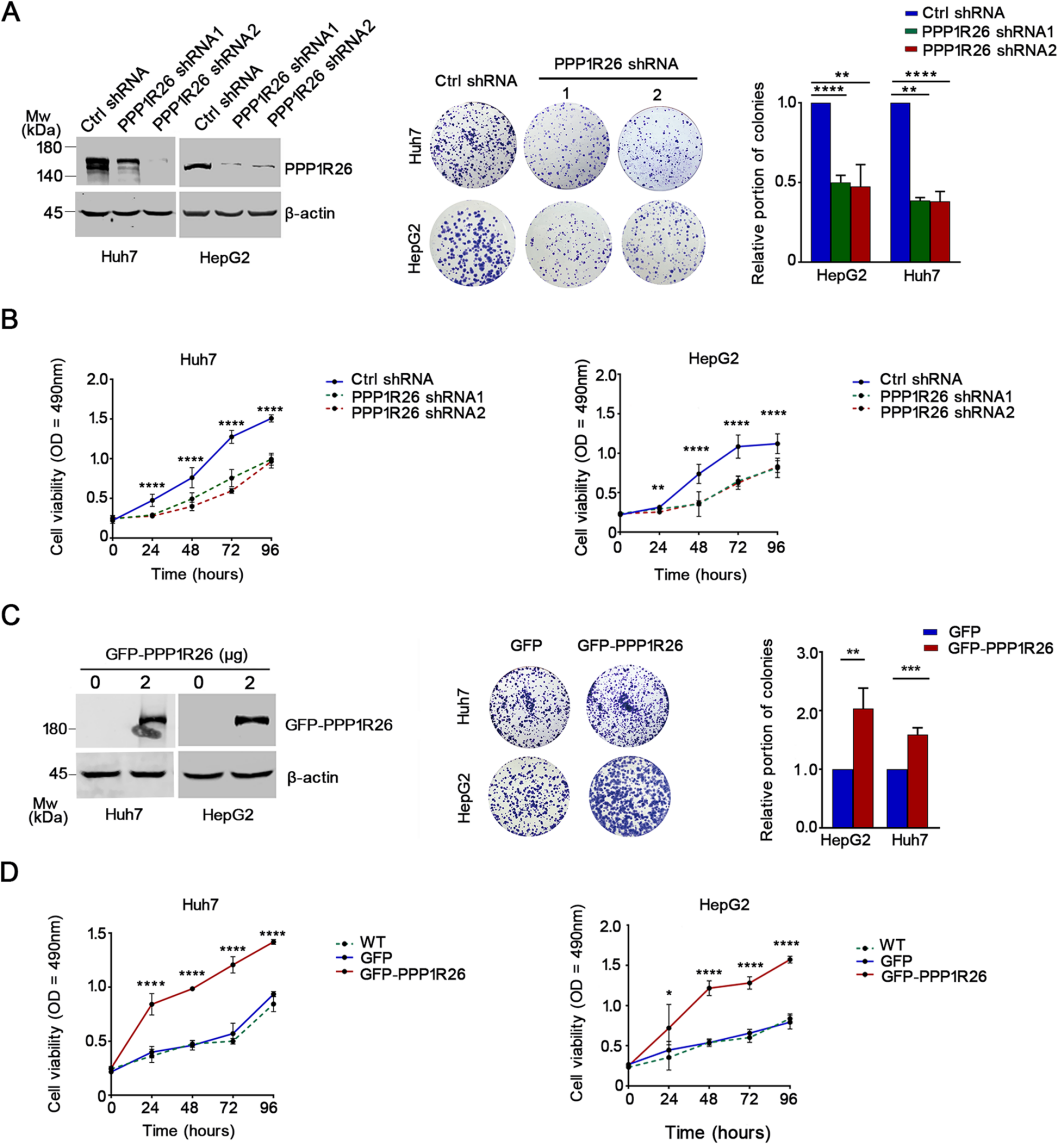
PPP1R26 promotes cell proliferation in HCC cells. **A** Expression of PPP1R26 was evaluated by Western blot in the indicated cells stably expressing shRNA (left). Beta-actin was used as a loading control. Colony formation was performed with indicated cells (middle). Colony formation data were summarized from three independent experiments in triplicates and showed in histograms (right). **B** Cell viability was evaluated in the indicated cells stably expressing shRNA using MTS assays. **C** GFP-PPP1R26 or GFP was transfected in Huh7 and HepG2 cells. Expression of GFP-PPP1R26 was evaluated by Western blot (left). Beta-actin was used as a loading control. Colony formation assays were performed (middle). Colony formation data were summarized from three independent experiments in triplicates and showed in histograms (right). **D** MTS was used to evaluate cell proliferation in Huh7 and HepG2 cells expressing GFP-PPP1R26 or GFP, respectively. Data information: In (**A-D**), data are presented as mean ± SD. Statistical significance was assessed using two-tailed t-tests (**C**) or one-way ANOVA with post hoc analysis LSD test (**A, B & D**). *P < 0.05, **P < 0.01, ***P < 0.001 and ****P < 0.0001

We then explored the function of PPP1R26 in HCC cell proliferation. Huh7, HepG2 and SMMC7721 cells were infected with lentiviruses expressing PPP1R26 shRNA to deplete PPP1R26 (Fig. 2A left, Supplementary Fig. 1A left). The colony formation assay revealed that knockdown of PPP1R26 decreased the proliferative capacity in Huh7, HepG2 and SMMC7721 cells (Fig. 2A middle & right, Supplementary Fig. 1B left & 1C). Accordingly, the growth curve plotted with the MTS assay showed that depletion of PPP1R26 decreased cell proliferation in these cells (Fig. 2B, Supplementary Fig. 1D left). To confirm these phenomena, GFP-PPP1R26 was ectopically expressed in Huh7, HepG2 and SMMC7721 cells (Fig. 2C left, Supplementary Fig. 1A right). GFP-PPP1R26 significantly increased the proliferative capacity in these cells evaluated by colony formation (Fig. 2C middle & right, Supplementary Fig. 1B right & 1C) and growth curve (Fig. 2D, Supplementary Fig. 1D right).

**Fig. 3:**
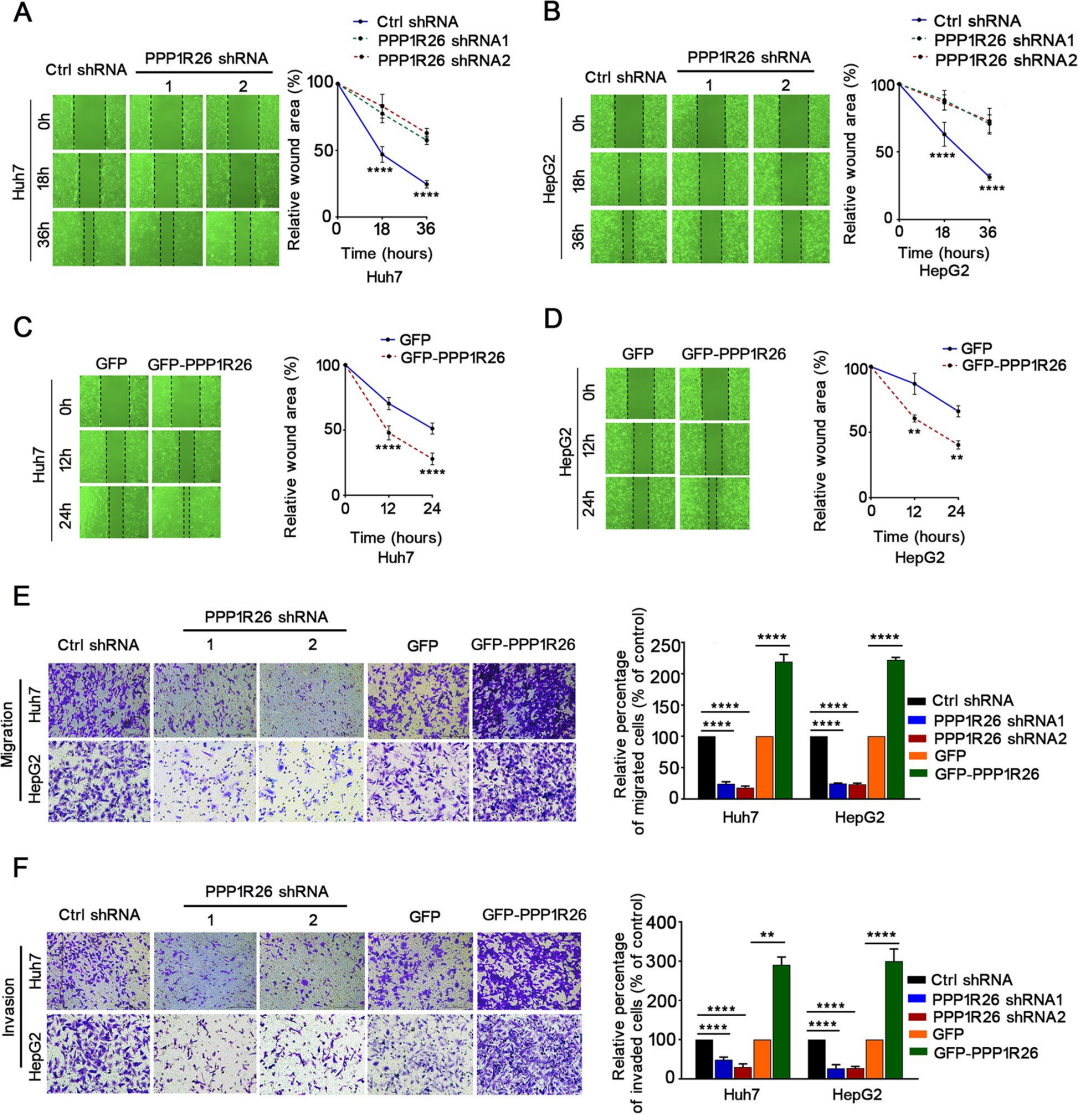
PPP1R26 promotes cell migration and invasion in HCC cells. **A** Wound-healing experiments were done in Huh7 cells with PPP1R26 depletion (left). Line charts of relative wound area are shown (right). **B** Wound-healing experiments were done in HepG2 with PPP1R26 depletion (left). Line charts of relative wound area are shown (right). **C** Wound-healing experiments in Huh7 transfected with GFP or GFP-PPP1R26 (left). Line charts of relative wound area are shown (right). **D** Wound-healing experiments in HepG2 transfected with GFP or GFP-PPP1R26 (left). Line charts of relative wound area are shown (right). **E** Migration transwell experiments in Huh7 and HepG2 cells after knockdown of PPP1R26 or overexpression of GFP-PPP1R26 (left). Histogram analyses of relative migrated cell counts are shown (right). The migration cells were counted in five random fields under microscope. **F** Invasion transwell experiments in Huh7 and HepG2 cells after knockdown of PPP1R26 or overexpression of GFP-PPP1R26 (left). Histogram analyses of relative invasive cell counts are shown (right). The invasive cells were counted in five random fields under microscope. Data information: In (**A-F**), data are presented as mean ± SD. Statistical significance was assessed using two-tailed t-tests (**C, D, E & F**) or one-way ANOVA with post hoc analysis LSD test (**A, E & F**). **P < 0.01 and ****P < 0.0001

Since PPP1R26 is tightly related to microscopic vascular metastasis in HCC patients, we evaluated the effect of PPP1R26 expression on cell migration and invasion. Cell migration and invasion were attenuated by the depletion of PPP1R26 in Huh7, HepG2 and SMMC7721 cells (Fig. 3A, B, E & F, Supplementary Fig. 1E & G), while the migration and invasion were enhanced by ectopic expression of GFP-PPP1R26 (Fig. 3 C, D, E & F, Supplementary Fig. 1F & G). Collectively, our findings indicate that PPP1R26 promotes cell proliferation, migration and invasion in HCC cells.

### PPP1R26 binds to PTBP1 to activate the alternative splicing of *PKM2*

**Fig. 4:**
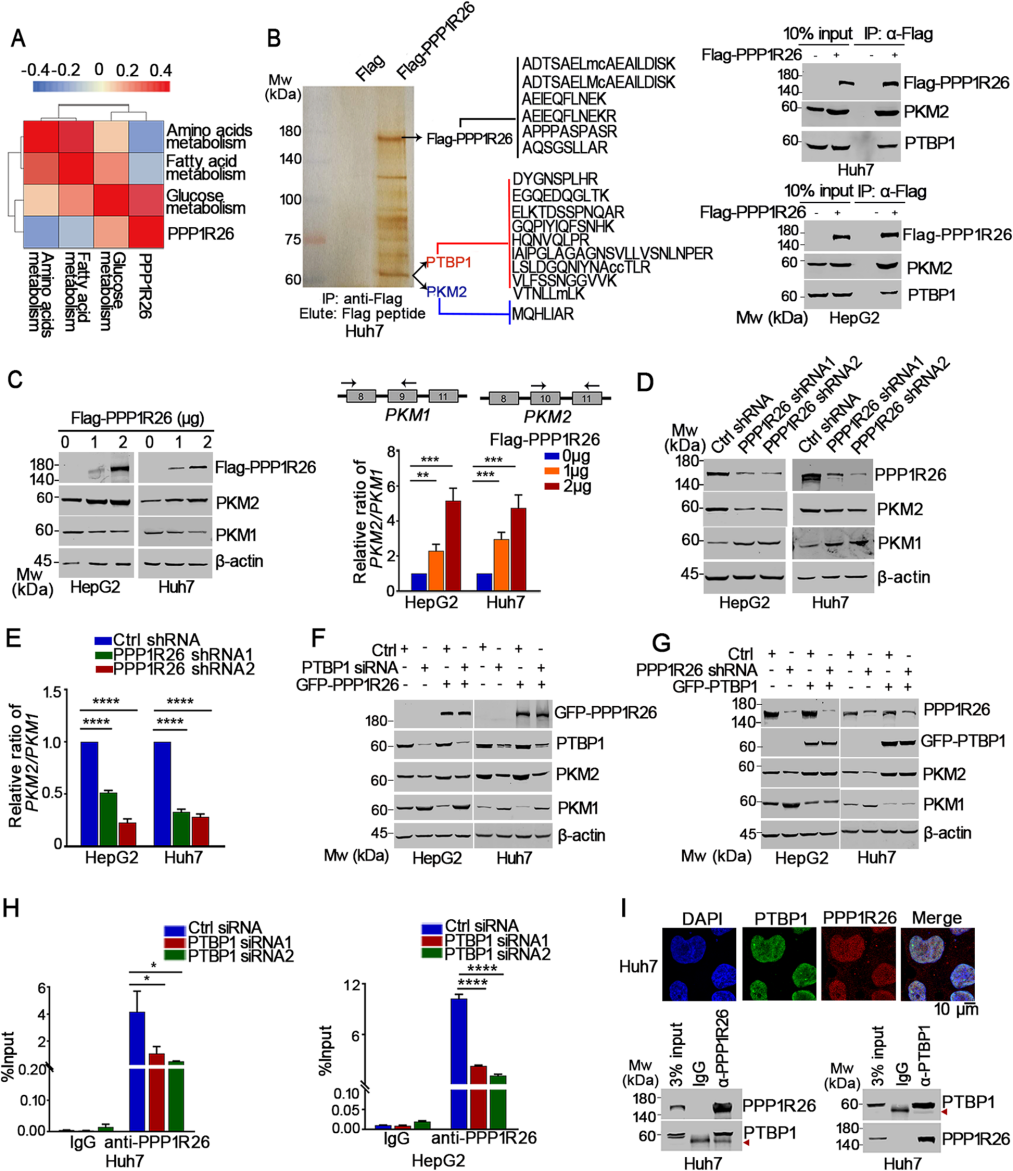
PPP1R26 is a glycolysis-associated protein and interacts with PTBP1 to control PKM2 splicing. **A** The correlation heatmap between expression of PPP1R26 and metabolic level in TCGA LIHC cohort. **B** Huh7 cells were transfected with Flag-PPP1R26 or Flag, and immunoprecipitation was performed with anti-Flag antibody on the cell extracts. PPP1R26-binding proteins were resolved by SDS-PAGE, detected by silver staining and analyzed by mass spectrometry (left). Flag or Flag-PPP1R26 was transfected into Huh7 and HepG2 cells, and immunoprecipitation was performed with anti-Flag antibody. Western blot was done with the indicated antibodies (right). **C** HCC cells were transfected with different dosages of Flag-PPP1R26. Western blot was done with the indicated antibodies (left). *PKM2*/*PKM1* mRNA levels were evaluated by RT-qPCR when Flag-PPP1R26 or Flag was expressed in HepG2 and Huh7 cells (right). **D** PPP1R26 was depleted by shRNA and Western blot was performed to evaluate the expression of PPP1R26, PKM1 and PKM2. **E** *PKM2*/*PKM1* mRNA levels were evaluated by RT-qPCR when PPP1R26 was depleted by shRNA in HepG2 and Huh7 cells (right). **F** Western blot analysis of indicated protein levels when GFP-PPP1R26 was expressed in the PTBP1-depleted HCC cells. **G** Western blot analysis of indicated protein levels in HepG2-shPPP1R26 cells or Huh7-shPPP1R26 cells transfected with GFP-PTBP1. **H** Huh7 and HepG2 cells were transfected with indicated PTBP1 siRNAs. Immunoprecipitation was performed with anti-PPP1R26 antibody. The immunoprecipitated RNA was submitted to RT-qPCR to evaluate the enrichment of *PKM* fragment flanking exon 9 by PPP1R26. The RNA enrichment was determined relative to the non-targeting IgG control. **I** Immunofluorescence staining was performed with anti-PPP1R26 and anti-PTBP1 antibodies. Nuclei were stained with DAPI (upper). Immunoprecipitation was performed with anti-PPP1R26 or anti-PTBP1 antibodies on Huh7 cell lysates. Rad arrows point to the heavy chain of IgG (lower). Data information: In (**C, E & H**), data are presented as mean ± SD. Statistical significance was assessed using one-way ANOVA with post hoc analysis LSD test. *P < 0.05, **P < 0.01, ***P < 0.001 and ****P < 0.0001.

Metabolic reprogramming is one of the characteristics of cancer, facilitating malignant activities in cancer cells. We therefore analyzed the metabolic levels including glucose, fatty acid and amino acid metabolisms in 374 HCC samples according to the TCGA LIHC cohort (Supplementary Table 5). The correlation heatmap showed that the expression of PPP1R26 is positively related to glucose metabolism, whereas negatively related to fatty acid and amino acid metabolisms (Fig. 4A, Supplementary Fig. 2A), indicating that PPP1R26 might activate glucose metabolism in HCC. To determine if PPP1R26 participates in glucose metabolism, we identified PPP1R26-binding proteins by mass spectrometry analysis on the Flag-PPP1R26-specific immunocomplex in Huh7 cells (Fig. 4B, left). Among the Flag-PPP1R26-binding proteins, a vital rate-limiting enzyme of glycolysis, PKM2 and a splicing factor of PKM2, PTBP1 raised our particular interest due to their essential roles in glycolysis (Fig. 4B, left). The binding of PTBP1 and PKM2 with Flag-PPP1R26 was further confirmed by immunoprecipitation in Huh7 and HepG2 cells (Fig. 4B right).

To determine if PPP1R26 is involved in the regulation of *PKM* splicing, we firstly evaluated the PKM2 and PKM1 expression levels when Flag-PPP1R26 was ectopically expressed. PKM2 protein level was upregulated, while PKM1 level was downregulated by Flag-PPP1R26 in HCC cells (Fig. 4C left, Supplementary Fig. 2B left). Further, RT-qPCR was used to evaluate the mRNA levels of *PKM1* and *PKM2*. Flag-PPP1R26 caused a dose-dependent increase of the ratio of *PKM2/PKM1* mRNA (Fig. 4C right, Supplementary Fig. 2B right). In contrast, PKM2 protein level was downregulated, while PKM1 level was upregulated when PPP1R26 was depleted in HCC cells (Fig. 4D, Supplementary Fig. 2C left). Accordingly, depletion of PPP1R26 repressed the ratio of *PKM2/PKM1* mRNA (Fig. 4E, Supplementary Fig. 2C right). Thus, we demonstrate that PPP1R26 promotes PKM2 expression by facilitating *PKM2* splicing.

The previous study has demonstrated that PTBP1 allows for the recognition of exon 9 by the splicing machinery and disrupts the intronic structures favorable for exon 10 inclusion, leading to increased expression of PKM2 [16] (Supplementary Fig. 2D). To determine if the PPP1R26-mediated mRNA splicing of *PKM2* is dependent on PTBP1, we depleted PTBP1 in the GFP-PPP1R26-expressed cells. GFP-PPP1R26 failed to regulate expression levels of PKM2 and PKM1 in the absence of PTBP1 (Fig. 4F). Meanwhile, the PPP1R26 depletion-induced expression alteration of PKM2 and PKM1 were reversed by GFP-PTBP1 (Fig. 4G), suggesting that PPP1R26 possibly promote PKM2 expression dependent on PTBP1. To further confirm the involvement of PPP1R26 in the *PKM* splicing, we performed RIP assays in HCC cells. PPP1R26 bound the intronic fragments flanking *PKM* exon 9 in RIP assays, and the intronic fragments flanking *PKM* exon 9 were less captured by PPP1R26 when PTBP1 was depleted (Fig. 4H). Additionally, ectopic GFP-PPP1R26 enhanced the binding of PTBP1 on the intronic fragments flanking *PKM* exon 9 (Supplementary Fig. 2E). These results indicated that PPP1R26 promotes *PKM2* splicing at least partially dependent on PTBP1. We further show that PPP1R26 endogenously binds to PTBP1 in the nucleus by immunofluorescent staining (Fig. 4I upper, Supplementary Fig. 2F left) and immunoprecipitation (Fig. 4I lower, Supplementary Fig. 2F right). Collectively, we demonstrate that PPP1R26 binds PTBP1 to promote *PKM2* splicing.

### PPP1R26 promotes glycolysis in HCC cells and *in vivo*

**Fig. 5:**
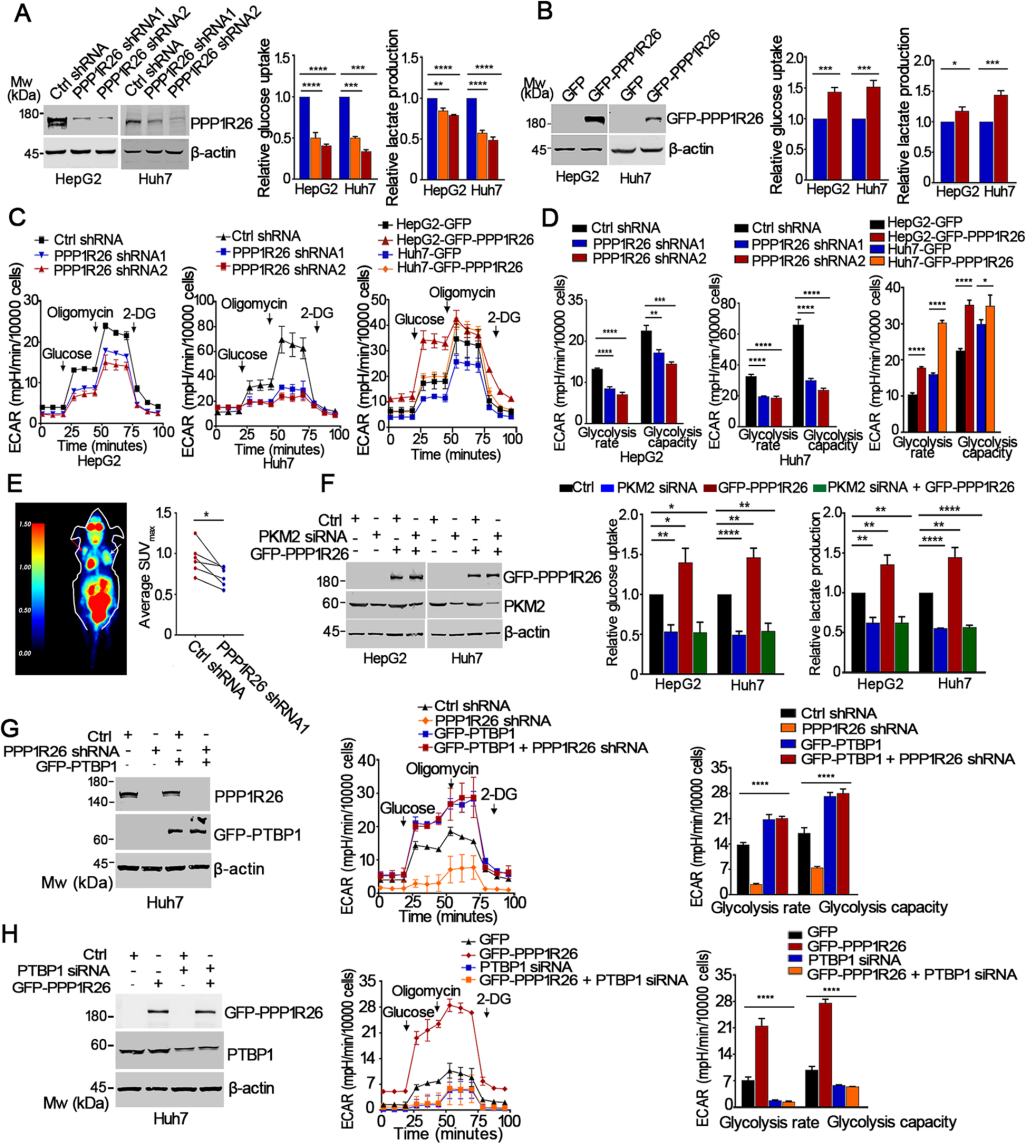
PPP1R26 promotes glycolysis in HCC cells and *in vivo*. **A** Expression of PPP1R26 was evaluated by Western blot in the HepG2 and Huh7 stably expressing shRNAs (left). Beta-actin was used as a loading control. Glucose uptake and lactate production in PPP1R26-depleted HepG2 and Huh7 cells were detected (middle & right). **B** GFP-PPP1R26 or GFP was transfected into HepG2 and Huh7 cells. Expression of GFP-PPP1R26 was evaluated by Western blot (left). Beta-actin was used as a loading control. Glucose uptake and lactate production in HepG2 and Huh7 were detected (middle & right). **C** The extracellular acidification rate (ECAR) was evaluated in HepG2-PPP1R26 shRNA (left) and Huh7-PPP1R26 shRNA (middle) cells treated with glucose, oligomycin, and 2-deoxyglucose (2-DG), respectively. HepG2 and Huh7 cells were transfected with GFP or GFP-PPP1R26 (right). **D** The histogram analysis of glycolysis rate (ECAR after glucose injection) and glycolysis capacity (ECAR after oligomycin injection) in (**C**). **E** Huh7-PPP1R26 shRNA cells were injected subcutaneously into the left (rad arrow) and Huh7-Ctrl shRNA cells in the right (white arrow) flanks of BALB/C nude mice. PET/CT imaging was performed. Representative PET photographs of animals were shown (left). Glucose uptake in the tumor was evaluated by the average SUV_max_ (right). **F** Western blot analysis of indicated protein levels when GFP-PPP1R26 was expressed in the PKM2-depleted HCC cells (left). Glucose uptake and lactate production were detected when GFP-PPP1R26 was expressed in the PKM2-depleted HCC cells (middle & right). **G** Expression of PPP1R26 and GFP-PTBP1 were evaluated by Western blot in the indicated cells (left). Beta-actin was used as a loading control. ECAR was evaluated in PPP1R26-depleted Huh7 cells after GFP-PTBP1 was expressed (middle). The histogram analysis of ECAR results (right). **H** Expression of GFP-PPP1R26 and PTBP1 were detected by Western blot in the indicated cells (left). Beta-actin was used as a loading control. ECAR was evaluated in PTBP1-depleted Huh7 cells when GFP-PPP1R26 was expressed (middle). The histogram analysis of ECAR results (right). Data information: In (**A-H**), data are presented as mean ± SD. Statistical significance was assessed using two-tailed t-tests **(B, D & F)** or one-way ANOVA with post hoc analysis LSD test **(A, D, G & H)**. *P < 0.05, **P < 0.01, ***P < 0.001 and ****P < 0.0001

**Fig. 6:**
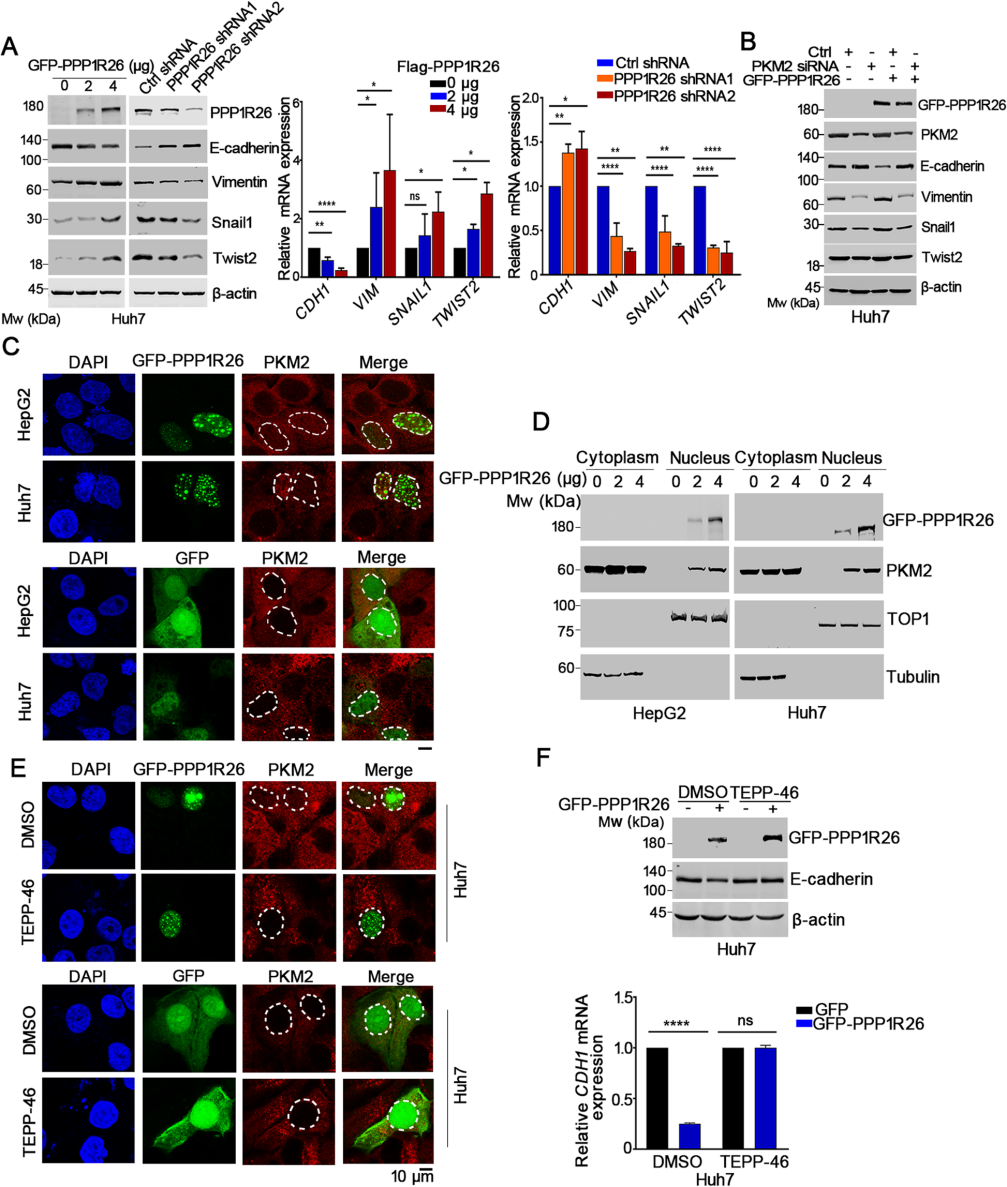
Overexpressed PPP1R26 induces accumulation of nuclear PKM2 and promotes EMT in HCC cells dependent on nuclear PKM2. **A** The expression of E-cadherin, Vimentin, Snail1 and Twist2 was determined by Western blot in the indicated cells (left). Relative mRNA levels of *CDH1, VIM, SNAIL1* and *TWIST2* were evaluated by RT-qPCR in the indicated cells (middle and right). Beta-actin was used as a loading control. **B** Western blot analysis of indicated protein levels when GFP-PPP1R26 was expressed in the PKM2-depleted Huh7 cells. **C** GFP-PPP1R26 (upper) or GFP (lower) was transfected into HepG2 and Huh7 cells. Cells were cultured under serum starvation and immunofluorescence staining was performed with anti-PKM2 antibody (red). DAPI was used to stain the nucleus. **D** HepG2 and Huh7 cells were transfected with GFP or GFP-PPP1R26 and cultured under serum starvation. Cytosolic and nuclear fractionations were prepared and the expression of PKM2 was determined by Western blot. TOP1 and Tubulin were used as the nuclear marker and cytoplasm marker, respectively. **E** Cells were treated with DMSO or TEPP-46 for 24 h after GFP-PPP1R26 (upper) or GFP (lower) was transfected. Immunofluorescence staining was done with anti-PKM2 (red). DAPI stained nuclei. Immunofluorescence images were taken using a confocal microscope. **F** Huh7 cells were treated with DMSO or TEPP-46 for 24 h after GFP-PPP1R26 was transfected. Whole cell extracts were used to evaluate the expression of GFP-PPP1R26 and E-cadherin by Western blot (upper). Relative mRNA levels of *CDH1* after treatment with DMSO or TEPP-46 for 24 h in GFP-PPP1R26 or GFP expressing Huh7 cells (lower). Data information: In (**A** & **F**), data are presented as mean ± SD. Statistical significance was assessed using one-way ANOVA with post hoc analysis LSD test (**A**) or two-tailed t-tests (**F**). **P < 0.01 and ****P < 0.0001. ns denotes no significance

We thereafter wanted to know if PPP1R26 regulates glycolysis in HCC cells. Glucose uptake and lactate production assays were performed. Knockdown of PPP1R26 led to a remarkable decrease in glucose uptake and lactate production in Huh7, HepG2 and SMMC7721 cells (Fig. 5A, Supplementary Fig. 3A). Accordingly, GFP-PPP1R26 significantly increased glucose uptake and lactate production in Huh7, HepG2 and SMMC7721 cells (Fig. 5B, Supplementary Fig. 3B). We then performed extracellular flux analysis to monitor real-time glucose-induced extracellular acidification rate (ECAR). The glycolysis rate and the glycolytic capacity were significantly attenuated by depletion of PPP1R26 and elevated by GFP-PPP1R26 (Fig. 5C & D, Supplementary Fig. 3C & D) in HCC cells. To further explore the function of PPP1R26 in glucose utilization *in vivo*, nude mice xenografts were established by subcutaneously injecting Huh7-PPP1R26-shRNA, Huh7-control-shRNA, SMMC7721-PPP1R26-shRNA or SMMC7721-control-shRNA cells. The uptake of glucose analog ^18^F-FDG was evaluated by PET imaging in the mouse xenografts. PPP1R26 depletion suppressed the glucose uptake as shown by the reduced ^18^F-FDG accumulation in the xenografts (Fig. 5E, Supplementary Fig. 3E). The SUV_max_ values in the tumors derived from Huh7-PPP1R26-shRNA cells (0.72 ± 0.12) were significantly lower than those in the tumors derived from the Huh7-control-shRNA1 cells (0.94 ± 0.19) (Fig. 6E right). Therefore, we demonstrate that PPP1R26 promotes glycolysis in HCC *in vivo*.

Furthermore, PPP1R26 depletion-reduced glycolysis was restored by GFP-PTBP1 (Fig. 5G, Supplementary Fig. 3F). GFP-PPP1R26-accelerated glycolysis was slowed down by depletion of PTBP1 (Fig. 5H, Supplementary Fig. 3G). These results demonstrate that PPP1R26 may possibly activates glycolysis by promoting *PKM2* splicing in a PTBP1-dependent mechanism in HCC cells.

### Overexpressed PPP1R26 promotes EMT by increasing the nuclear accumulation of PKM2

To determine the causative role of PP1R26 in cell migration and invasion, we evaluated EMT markers in HCC cells. GFP-PPP1R26 inhibited the protein and mRNA levels of E-cadherin and elevated the protein and mRNA levels of Vimentin, Snail1 and TWIST2 in a dose dependent manner. Depletion of PPP1R26 led to upregulation of E-cadherin and downregulation of Vimentin, Snail1 and TWIST2 at mRNA and protein levels, implying that PPP1R26 promotes EMT (Fig. 6A). PKM2 is reported to regulate EMT in tumors [31, 32], we then determined if PPP1R26 promotes EMT dependent on PKM2. GFP-PPP1R26 failed to downregulate E-cadherin, and upregulate Vimentin, Snail1 and Twist2 in PKM2-depleted cells (Fig. 6B), suggesting that PPP1R26 promotes EMT in a PKM2 dependent way.

Previous studies found that the nuclear PKM2 represses *CDH1* transcription [20]. We thereafter asked if upregulated PPP1R26 promotes EMT through regulating nuclear PKM2 in HCC cells. To answer this question, we expressed GFP-PPP1R26 and determined the cellular localization of PKM2 in Huh7, HepG2 and SMMC7721 cells. We showed that PKM2 localized in the nucleus in the GFP-PPP1R26 expressed cells under serum starvation condition, whereas it localized in the cytoplasm in the GFP-expressed cells (Fig. 6C, Supplementary Fig. 4A). To confirm this phenomenon, we evaluated the PKM2 level in the cellular fractionation when cells were transfected with GFP-PPP1R26 and serum starved. PKM2 accumulated in the nucleus only when GFP-PPP1R26 was expressed under serum starvation in Huh7, HepG2 and SMMC7721 cells (Fig. 6D, Supplementary Fig. 4B), indicating that upregulation of PPP1R26 induces the nuclear accumulation of PKM2.

Next, we wanted to know if PPP1R26 regulates EMT dependent on the nuclear accumulation of PKM2 in HCC cells. Since the PKM2-tetramer stabilizer TEPP-46 blocks the nuclear translocation of PKM2 [33], we treated cells with TEPP-46 and determined PKM2 localization. GFP-PPP1R26 failed to induce the nuclear accumulation of PKM2 under TEPP-46 treatment (Fig. 6E, Supplementary Fig. 4C). Notably, TEPP-46 suppressed the GFP-PPP1R26-induced EMT, as evidenced by the protein level of E-cadherin (Fig. 6F upper, Supplementary Fig. 4D upper) and the *CDH1* mRNA level (Fig. 6F lower, Supplementary Fig. 4D lower). Taken together, we demonstrate that upregulated PPP1R26 promotes EMT by increasing the nuclear accumulation of PKM2 to repress the transcription of *CDH1*.

### Upregulated PPP1R26 forms a complex with p-Ser37-PKM2 and TGIF2 to inhibit the transcription of *CDH1*

**Fig. 7:**
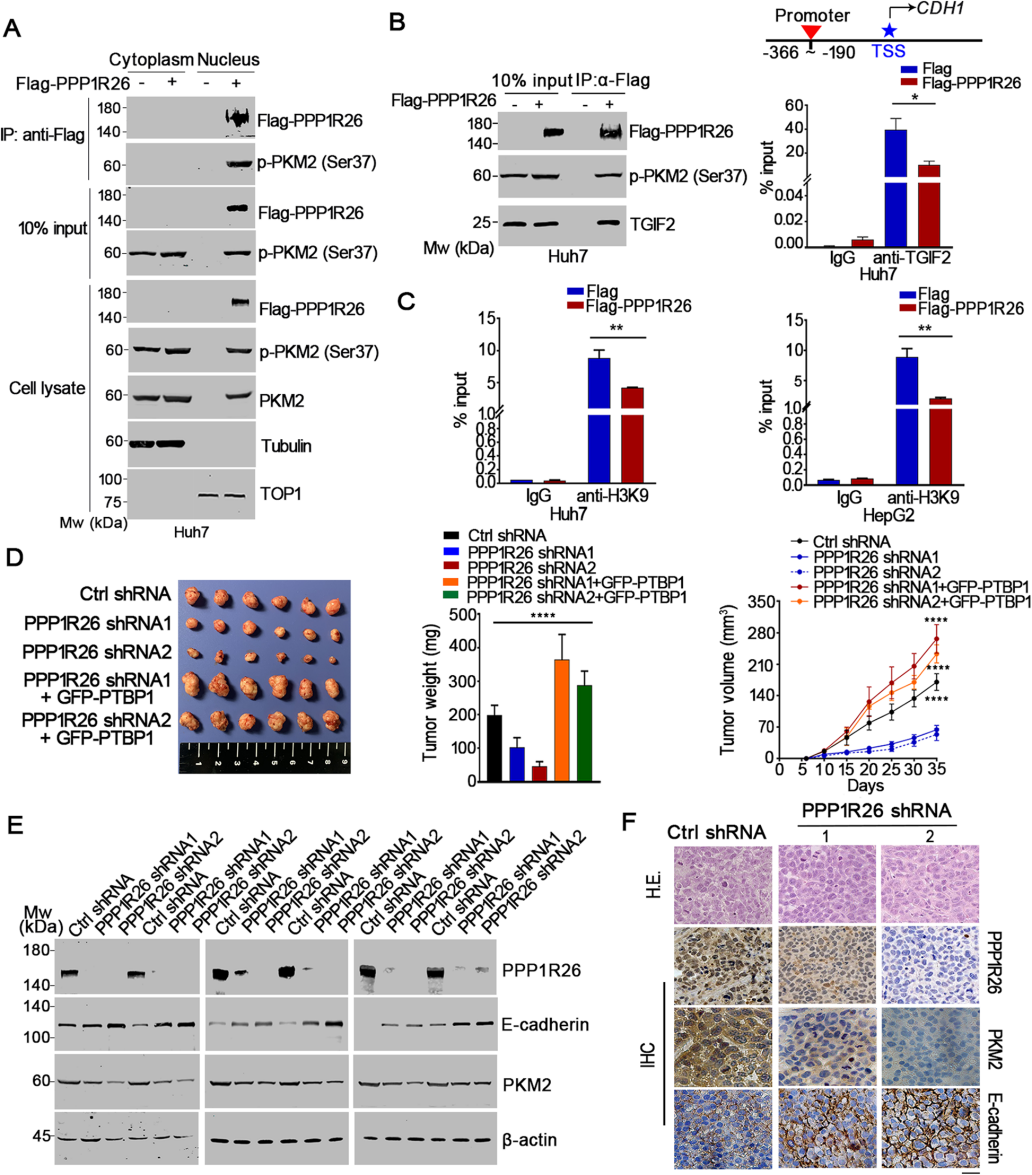
PPP1R26 promotes EMT in HCC cells by forming the PPP1R26-pSer37 PKM2-TGIF2 complex. **A** Huh7 cells were transfected with Flag or Flag-PPP1R26 and cultured under serum starvation. Immunoprecipitation was performed with an anti-Flag antibody on the cellular fractionation. The Flag-PPP1R26 and p-Ser37-PKM2 in the precipitates were determined by Western blot. **B** Huh7 cells were transfected with Flag or Flag-PPP1R26 and cultured under serum starvation. Immunoprecipitation was performed with an anti-Flag antibody. The Flag-PPP1R26, p-Ser37-PKM2 and TGIF2 in the precipitates were determined by Western blot (left). Schematic diagram showing the positions of primers designed to cover the promoter region of the *CDH1* gene (right, upper). Huh7 cells were transfected with Flag or Flag-PPP1R26. ChIP assays were performed with IgG or anti-TGIF2 antibody followed by qPCR to amplify the *CDH1* promoter region (right, lower). **C** Huh7 cells was transfected with Flag or Flag-PPP1R26. ChIP assays were performed with IgG and anti-acetylated H3K9 antibody followed by qPCR as described in **B**. **D** Huh7-PPP1R26-shRNA cells were subcutaneously implanted into nude mice. Tumors were dissected at the end of the experiment (left). Tumor weights were evaluated and shown in the middle. Tumor volumes were measured every 5 days during the experiments as shown in the right. **E** Proteins extracted from the xenografts were subjected to Western blotting probed with anti-PPP1R26, anti-PKM2 and anti-E-cadherin antibodies. Beta-actin was used as a loading control. **F** IHC was performed with anti-PPP1R26, anti-PKM2 and anti-E-cadherin antibodies on the paraffin-embedded tissue sections of xenografts (right). Data information: In (**A-D**), data are presented as mean ± SD. Statistical significance was assessed using two-tailed t-tests (**B & C**), one-way ANOVA with post hoc analysis LSD test or Kruskal-Wallis test (**D**). *P < 0.05, **P < 0.01 and ****P < 0.0001

It was previously found that only the monomeric or dimeric forms of PKM2 could translocate into the nucleus due to the Ser37 phosphorylation (p-Ser37-PKM2) [34, 35]. We then determined if PPP1R26 binds to p-Ser37-PKM2 in the nucleus. We performed immunoprecipitation with anti-Flag antibody on the cellular fractionation when Flag or Flag-PPP1R26 was expressed under serum starvation conditions. We showed that Flag-PPP1R26 binds to p-Ser37-PKM2 in the nucleus (Fig. 7A, Supplementary Fig. 5A). Additionally, the cytoplasmic p-Ser37-PKM2 level also increased in the Flag-PPP1R26 expressed cells due to the increased total level of PKM2. Thus, our data indicated that upregulated PPP1R26 bound to p-Ser37-PKM2 and increased the nuclear accumulation of PKM2 in HCC cells.

TGIF2 is a transcriptional factor that binds to *CDH1* promoter to activate *CDH1* transcription [20]. Under EGF stimulation, PKM2 translocates into the nucleus and binds to TGIF2 to loosen the binding of TGIF2 with *CDH1* promoter, which allows the recruitment of HDAC3 and subsequent histone H3 deacetylation, thus resulting in the suppression of *CDH1* transcription [20]. To determine if PPP1R26 forms a complex with p-Ser37-PKM2 and TGIF2, we performed IP experiments. We show that Flag-PPP1R26 interacts with the pSer37-PKM2 and TGIF2 (Fig. 7B left, Supplementary Fig. 5B upper). Importantly, ChIP qPCR assay showed that Flag-PPP1R26 dramatically inhibits the binding of TGIF2 with *CDH1* promoter (Fig. 7B right, Supplementary Fig. 5B lower), demonstrating that upregulated PPP1R26 inhibits the transcription of *CDH1* by forming a nuclear complex with p-Ser37-PKM2 and TGIF2. We further determined the acetylation levels of histone H3 in *CDH1* promoter by ChIP qPCR assay as described previously [20]. We show that the acetylation level of H3K9 in the *CDH1* promoter decreased when Flag-PPP1R26 was ectopically expressed in HCC cells (Fig. 7C), demonstrating that PPP1R26 forms a complex with PKM2 and TGIF2 to loosen TGIF2-*CDH1* promoter binding, leading to suppression of *CDH1* transcription.

### PPP1R26 depletion attenuates tumorigenesis and metastatic ability

To further substantiate the function of PPP1R26 in hepato-carcinogenesis *in vivo*, nude mice xenografts were established by subcutaneously injecting Huh7-PPP1R26-shRNA or Huh7-control-shRNA cells. PPP1R26 depletion inhibited tumor growth (Fig. 7D left). The size and weight of tumors derived from the Huh7-PPP1R26-shRNA cells decreased compared with those derived from the Huh7-control-shRNA cells (Fig. 7D middle & right). Western blot and IHC revealed that the absence of PPP1R26 leads to downregulation of PKM2 and upregulation of E-cadherin in the xenografts *in vivo* (Fig. 7E & F), suggesting that depletion of PPP1R26 attenuates tumorigenesis and metastatic ability *in vivo*. This is further supported by the nude mice xenografts established by subcutaneously injecting SMMC7721-PPP1R26-shRNA or SMMC7721-control-shRNA cells (Supplementary Fig. 5C & D).

### Depletion of PPP1R26-reduced tumorigenesis and metastatic ability is restored by overexpression of PTBP1 *in vivo*

**Fig. 8:**
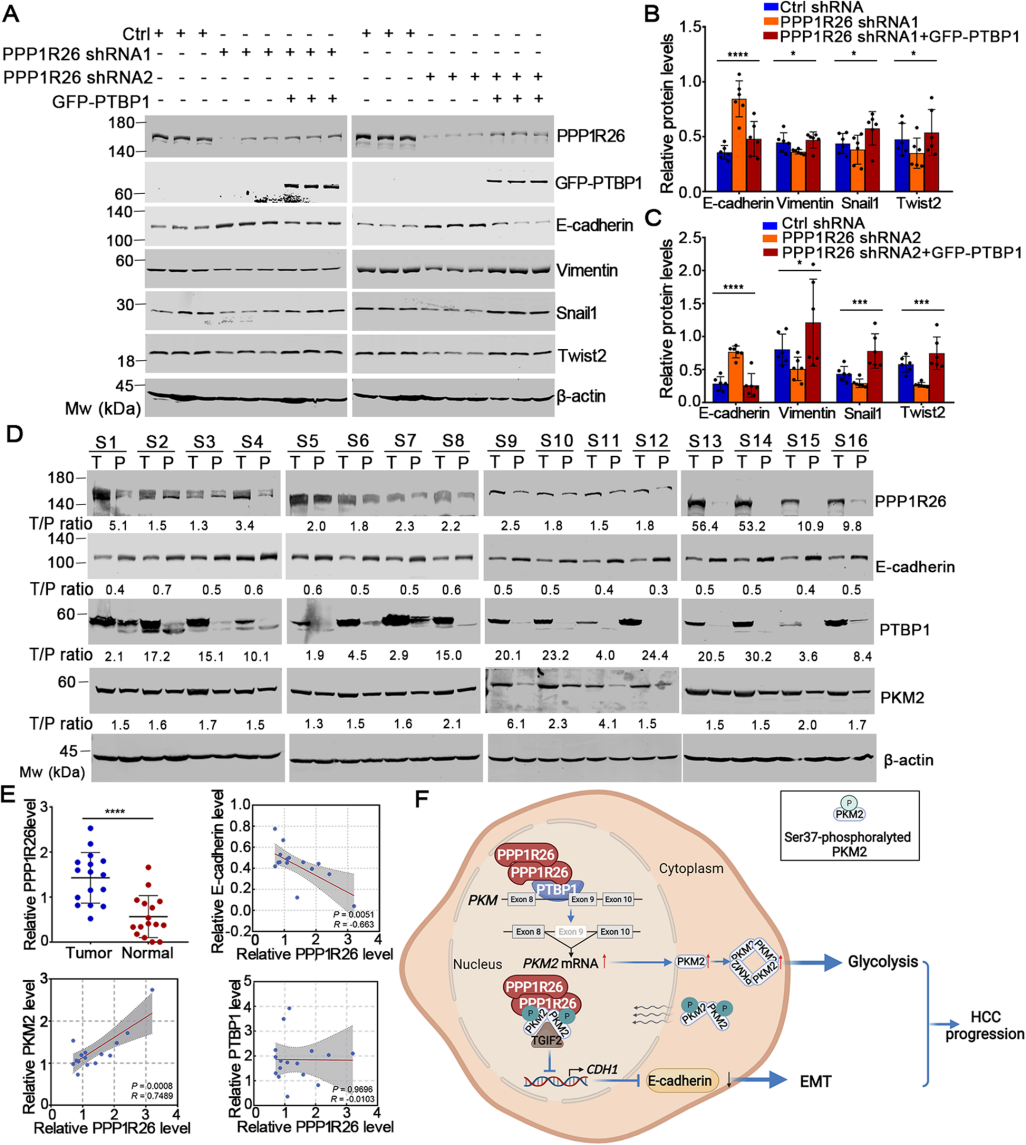
PPP1R26 regulates the expression of EMT markers and PKM2 to promote HCC progression. **A** Proteins extracted from the xenografts were subjected to Western blotting probed with anti-PPP1R26, anti-GFP, anti-E-cadherin, anti-Vimentin, anti-Snail1 and anti-Twist2 antibodies. **B**-**C** Densitometry scanning analyses of the E-cadherin, Vimentin, Snail1 and Twist2 bands standardized to beta-actin are summarized and shown. **D** Western blot analysis of PPP1R26, E-cadherin, PTBP1, PKM2 and beta-actin expression in 16 paired HCC tissues. T denotes tumor tissues and P represents para-tumor non-tumorous tissues. **E** The correlation among PPP1R26 protein level with E-cadherin, PTBP1 and PKM2 protein level in the human HCC tissues was plotted by GraphPad. **F** A working model illustrating that PPP1R26 promotes HCC progression by simultaneously regulating glycolysis and EMT. Data information: In (**B, C & E**), data are presented as mean ± SD. Statistical significance was assessed using one-way ANOVA with post hoc analysis LSD test (**B & C**) or two-tailed t-tests (**E**). *P < 0.05, ***P < 0.001 and ****P < 0.0001

To investigate if overexpression of PTBP1 could restore the tumorigenesis and metastatic ability reduced by knockdown of PPP1R26 *in vivo*, nude mice xenografts were established by subcutaneously injecting Huh7-PPP1R26-shRNA1 + GFP-PTBP1 or Huh7-PPP1R26-shRNA2 + GFP-PTBP1 cells (Fig. 7D left). GFP-PTBP1 restored the tumor volume and tumor weight reduced by PPP1R26-shRNA (Fig. 7D middle & right), revealing that overexpression of PTBP1 restores the tumorigenesis reduced by PPP1R26 depletion. We next determined the expression levels of EMT markers (E-cadherin, Vimentin, Snail1, Twist2) in the nude mice xenografts. The upregulated E-cadherin in PPP1R26-shRNA1 or PPP1R26-shRNA2 cells was lowered by GFP-PTBP1 *in vivo*. The downregulation of Vimentin, Snail1 and Twist2 were restored by GFP-PTBP1 *in vivo* (Fig. 8A, B & C, Supplementary Fig. 6). These results demonstrated that overexpression of PTBP1 restores the PPP1R26 depletion-reduced tumorigenesis and metastatic ability *in vivo*.

### PPP1R26 expression is correlated with PKM2 and E-cadherin in human HCC tissues

Finally, we determined the protein levels of PPP1R26, E-cadherin, PTBP1 and PKM2 in 16 cases of cancerous and paired non-cancerous human HCC tissues by immunoblotting (Fig. 8D). The expressions of PPP1R26, PTBP1 and PKM2 were upregulated in 93.8% (15/16), 100% (16/16) and 93.8% (15/16) in human HCC tumor tissues compared with the non-cancerous tissues, respectively (Fig. 8E). Meanwhile, the expression of E-cadherin was downregulated in 75% (12/16) of human HCC tumor tissues. The protein levels of PPP1R26 are positively correlated with the levels of PKM2, and are negatively correlated with the expression of E-cadherin in HCC tissues from patients (Fig. 8E). Collectively, our data confirmed that PPP1R26 promotes HCC progression by activating glycolysis and EMT (Fig. 8F).

## Discussion

Cancer cells display an enhanced glycolytic phenotype in tumor progression [36]. Moreover, the increased glucose uptake, glycolysis flux, a more acidic tumor microenvironment and mitochondrial dysfunction activate and maintain the metastatic potential in cancer cells [37–39]. Thus, elucidation of molecular mechanisms underlying the crosslink between glycolysis and metastasis could contribute to attenuating tumor progression. In the present study, we have explored how PPP1R26 concurrently modulates glycolysis and EMT to promote HCC progression.

We first found that PPP1R26 was upregulated in HCC and the upregulated expression of PPP1R26 was significantly related to the BCLC stage, microscopic vascular invasion, recurrence and prognosis of the patients. We then demonstrated that PPP1R26 promotes proliferation, migration and invasion in HCC cells. Furthermore, depletion of PPP1R26 by shRNA dramatically inhibits tumor growth in mouse xenografts *in vivo,* demonstrating that small molecules targeting PPP1R26 such as siRNA or chemical compound may provide a potential therapeutic strategy in the HCC treatment.

To unravel the function of PPP1R26 in cancer cell metabolism, we performed bioinformatics analysis using the TCGA LIHC dataset. The correlation heatmap showed that PPP1R26 is positively correlated with glucose metabolism. Among the PPP1R26-binding proteins, PKM2 is an essential enzyme controlling glucose metabolism. We therefore set out to explore the function of PPP1R26 in glycolysis.

The present study showed that PPP1R26 promotes glucose uptake and lactate production in HCC cells, indicating that PPP1R26 increases glycolysis. During the glycolysis process, the conversion of pyruvate to lactate causes a net production of protons into the extracellular medium, resulting in an acidic medium. Thus, we used the XFe24 instrument to measure the acidification rate described previously as ECAR to determine glycolysis rate and capacity in HCC cells [40]. We showed that PPP1R26 enhances the glycolysis rate and capacity in HCC cells. Targeting PPP1R26 reduces the glycose metabolism and the acidification rate in HCC to devoid the favorable condition for tumor proliferation and metastasis.

The increased glucose consumption in cancer cells forms the basis of tumor imaging by PET. In clinical, ^18^F-FDG is used to trace the glucose uptake in cancer cells and becomes the critical clinical tool for staging and assessing cancer recurrence [41]. In the present study, we used PET imaging to evaluate the effect of PPP1R26 on glucose uptake in mouse xenografts *in vivo*. Depleting PPP1R26 by shRNA significantly decreased ^18^F-FDG accumulation in the xenografts derived from HCC cells, demonstrating that targeting PPP1R26 reduces glucose uptake and metabolism *in vivo*.

Given that PKM2 plays a vital role in glycolysis, we evaluated the effect of PPP1R26 on PKM2 expression. We found that PPP1R26 upregulates PKM2 expression by facilitating splicing of *PKM2*. PTBP1 acts as a splicing repressor of *PKM1* and promotes tumorigenesis. Mechanistically, PTBP1 binds the downstream pre-mRNA or forms RNA loop between high-affinity sites, thereby blocking access of splicing factors to their binding sites on the pre-mRNA [19]. The present study showed that endogenous PPP1R26 bound to PTBP1 and the intronic fragments flanking exon 9 of the *PKM* pre-mRNA to enhance *PKM2* splicing. Importantly, PPP1R26 promotes *PKM2* splicing and expression in a PTBP1-dependent manner. ECAR experiments also showed that PPP1R26 promotes glycolysis rate and capacity in HCC cells in a PTBP1-dependent way. Collectively, we demonstrate that PPP1R26 accelerates glycolysis through binding to PTBP1 to promote PKM2 expression in HCC cells. PTBP1 is known to be phosphorylated at multiple sites by mass spectrometry analysis [42]. PKA phosphorylates PTBP1 at Ser16 to promote PTBP1 nuclear export [43]. However, the particular kinase and the role of PTBP1 phosphorylation have not been defined. Thus, if PPP1R26 regulates the phosphorylation of PTBP1 to control its splicing capability needs further study.

Since PPP1R26 promotes cell migration, invasion and EMT in HCC cells, we further unraveled the involved mechanism. Previously study demonstrated that nuclear PKM2 binds to TGIF2 to loosen the binding of TGIF2 with *CDH1* promoter, which allows the recruitment of HDAC3 to the *CDH1* promoter, resulting in the deacetylation of histone H3 and suppression of *CDH1* transcription when TGF-β or EGF are added [20]. Our study shows that overexpressed PPP1R26 increases the nuclear accumulation of PKM2 under serum starvation conditions, indicating that upregulated PPP1R26 might control PKM2 function in the nucleus without TGF-β or EGF stimulation. We further show that overexpressed PPP1R26 forms a PPP1R26-pSer37-PKM2-TGIF2 complex to inhibit the binding of TGIF2 with *CDH1* promoter, and the H3 acetylation level in the *CDH1* promoter decreased, leading to transcriptional repression of *CDH1*. These results are consistent with PPP1R26 up-regulation promoting metastasis and progression in HCC patients. We thus unravel a novel mechanism by which PPP1R26 promotes HCC metastasis via preventing TGIF2 from binding with *CDH1* promoter, thus inhibiting *CDH1* transcription.

Previous studies found that PKM2 translocates into the nucleus in response to the activated MEK/ERK pathway [34, 40]. MEK/ERK signaling pathway is generally activated in several types of human cancers, and its activation promotes tumor cell proliferation and survival [44]. ERK1/2 phosphorylates PKM2 at Ser37 and leads to cis-trans isomerization of PKM2 by PIN1 to expose the PKM2 NLS to importin α5, transporting PKM2 into the nucleus [34, 35]. Thus, Ser37 phosphorylation and isomerization of PKM2 are indispensable for PKM2 nuclear translocation. However, if pSer37-PKM2 controls transcription of *CDH1* is unknown. We showed that Flag-PPP1R26 increased the level of pSer37-PKM2, which might be due to the increase of the total level of PKM2. Importantly, Flag-PPP1R26 bound the Ser37-phosphorylated PKM2 and TGIF2, inhibiting the binding of TGIF2 with *CDH1* promoter and repressing *CDH1* transcription. Therefore, our study provides evidence for the function of pSer37-PKM2 in the *CDH1* transcription regulation.

PTBP1 is known to play in various cancers, such as colorectal cancer, renal cell cancer, breast cancer, glioma and liver cancer [45–48]. PTBP1 controls glycolysis, apoptosis, proliferation, tumorigenesis, invasion and migration in cancer [45, 48, 49]. In the present study, we found that the expressions of both PTBP1 and PPP1R26 were upregulated in human HCC tumor tissues. Accordingly, PKM2 expression was upregulated and E-cadherin was downregulated. Importantly, the upregulation of PPP1R26 was significantly correlated with PKM2 and E-cadherin. These data suggest that the upregulated PPP1R26 may collaborate with PTBP1 to promote the expression of PKM2 and inhibit the expression of E-cadherin in human HCC tissues *in vivo*. Additionally, we show that the overexpressed GFP-PTBP1 restored the impaired tumorigenesis and metastatic ability by depletion of PPP1R26 in mouse xenografts, further confirming that PPP1R26 may promote the tumorigenesis and metastasis in HCC via collaborating with PTBP1 *in vivo*.

Taken together, we demonstrated that PPP1R26 promotes glycolysis in HCC cells dependent on PTBP1. PPP1R26 binds to PTBP1 on the *PKM* pre-mRNA to promote *PKM2* splicing and expression. Meanwhile, pSer37-PKM2 increases upon PPP1R26-induced upregulation of PKM2 expression. Increased pSer37-PKM2 translocates into the nucleus and forms a complex with upregulated PPP1R26 and TGIF2, leading to transcriptional repression of *CDH1*. However, if PPP1R26 regulates PKM2 phosphorylation at Ser37 concerning the function of protein phosphatase1 requires further exploration.

## Conclusions

In summary, we demonstrate that PPP1R26 is a potential prognostic biomarker. PPP1R26 coordinates glycolysis and EMT through promoting *PKM2* splicing and nuclear accumulation of PKM2. Our study provides new insight into the understanding of crosslinking of glycolysis and EMT in the progression of HCC (Fig. 8F). Targeting PPP1R26 by small chemical molecules or siRNAs provides a therapeutic strategy for the HCC patients with upregulation of PPP1R26.

## Supplementary Information

**Additional file 1: Supplementary Figs. 1–6. Supplementary Tables. 1–5**.

## Data Availability

All publicly available data can be acquired from the corresponding web servers described in the Materials and methods. The data used and/or analyzed in the present study are available from the corresponding author on reasonable request.

## Abbreviations

HCC: Hepatocellular carcinoma
PPP1R26: Protein phosphatase 1 regulatory subunit 26
EMT: Epithelial-mesenchymal transition
PKM1: Pyruvate kinase M1
PKM2: Pyruvate kinase M2
PTBP1: Polypyrimidine tract binding protein 1
GFP: Green fluorescent protein
FDG: Fluorodeoxyglucose
ECAR: Extracellular acidification rate
PET: Positron emission tomography
CT: Computed tomography
shRNA: Short hairpin RNA
siRNA: Small interfering RNA
TCGA: The Cancer Genome Atlas
BCLC: Barcelona Clinic Liver Cancer
