# Supplementary material for: PPP1R26 drives hepatocellular carcinoma progression by controlling glycolysis and epithelial-mesenchymal transition
Source: J Exp Clin Cancer Res. 2022 Mar 15;41:101. doi: 10.1186/s13046-022-02302-8 (PMC8922775; doi:10.1186/s13046-022-02302-8)
Supplement: Supplementary file 1 — Additional file 1: Supplementary Figs. 1–6. Supplementary Tables. 1–5. [file 13046_2022_2302_MOESM1_ESM.docx]

**Supplementary Fig. 1-6.**

**Fig. S1.**


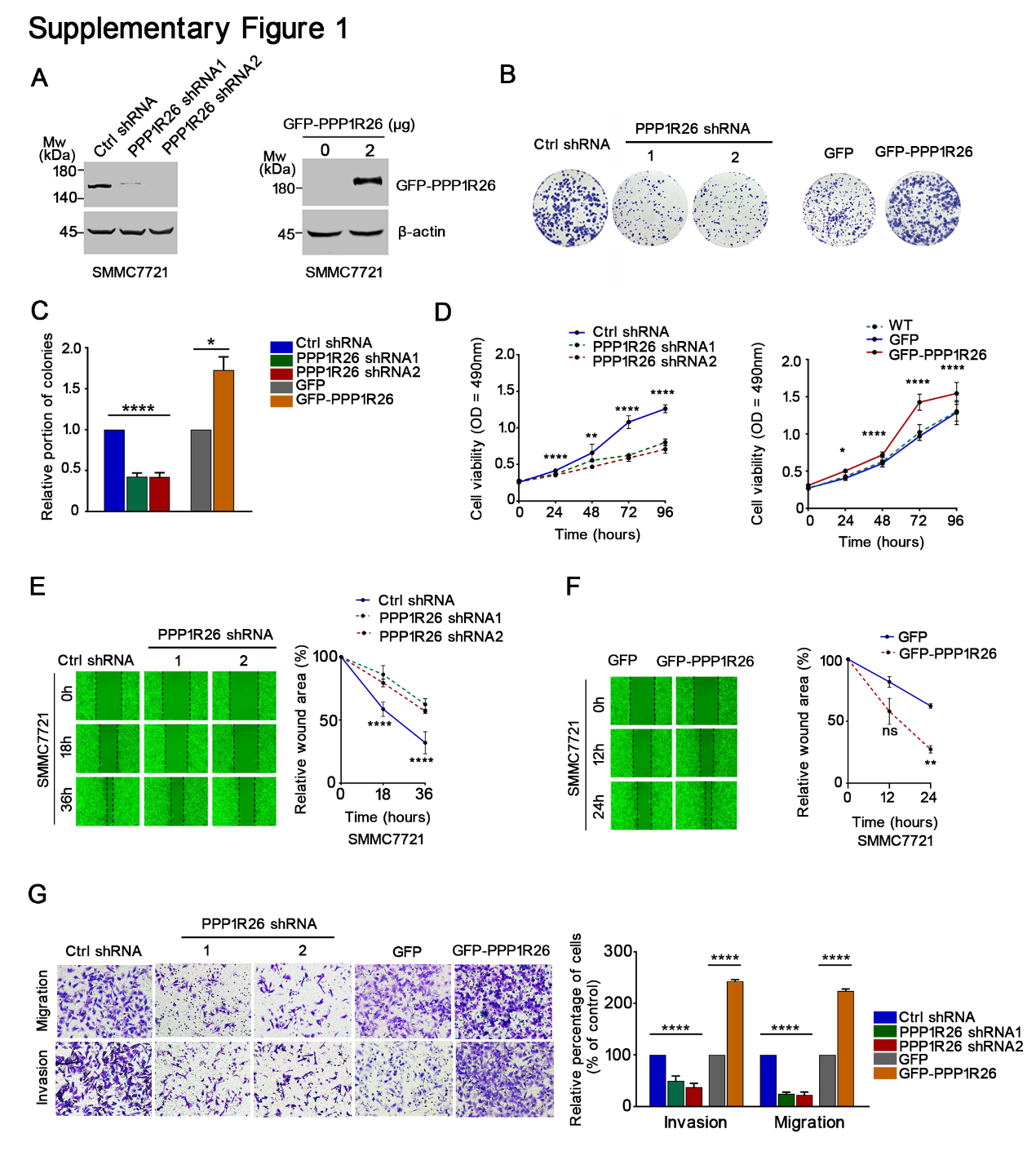


**Supplementary Fig. 1.** **PPP1R26 promotes cell proliferation, migration and invasion in SMMC7721 cells.** **(A)** Expression of PPP1R26 was evaluated by Western blot in the indicated cells stably expressing shRNA (left). GFP-PPP1R26 or GFP was transfected in SMMC7721 cells. Expression of GFP-PPP1R26 was evaluated by Western blot (right). β-actin was used as a loading control. **(B)** Colony formation was performed with indicated cells. **(C)** Colony formation data were summarized from three independent experiments in triplicates and showed in histograms. **(D)** Cell viability was evaluated in the indicated cells stably expressing shRNA using MTS assays (left). MTS was used to evaluate cell proliferation in SMMC7721 cells expressing GFP-PPP1R26 or GFP, respectively (right). **(E)** Wound-healing experiments were done in SMMC7721 cells with PPP1R26 depletion (left). Line charts of relative wound area are shown (right). **(F)** Wound-healing experiments in SMMC7721 transfected with GFP or GFP-PPP1R26 (left). Line charts of relative wound area are shown (right). **(G)** Migration transwell experiments in SMMC7721 cells after knockdown of PPP1R26 or overexpression of GFP-PPP1R26 (left, upper). Invasion transwell experiments in SMMC7721 cells after knockdown of PPP1R26 or overexpression of GFP-PPP1R26 (left, down). Histogram analyses of relative migrated and invasive cell counts are shown (right). The migration and invasion cells were counted in five random fields under microscope. Data information: In (C-G), data are presented as mean ± SD. Statistical significance was assessed using two-tailed t-tests (C, F & G) or one-way ANOVA with post hoc analysis LSD test (C, D, E & G). **P* < 0.05, ***P* < 0.01 and *****P* < 0.0001.

**Fig. S2.**


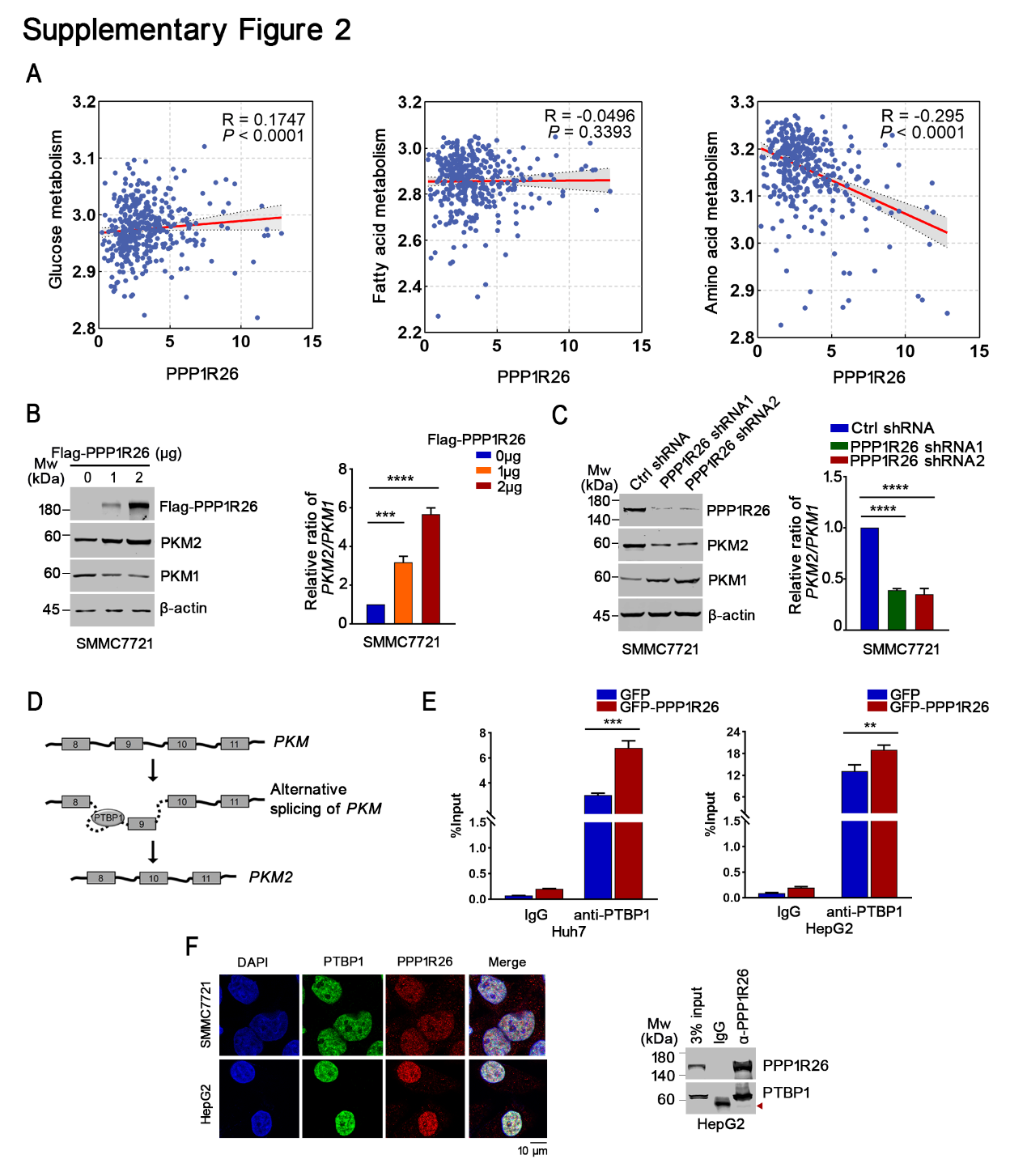


**Supplementary Fig. 2.** **PPP1R26 regulates glycolysis in SMMC7721 cells via PTBP1-PKM2 axis.** **(A)** The correlation analysis between the expression of PPP1R26 and ssGSEA score of glucose metabolism, fatty acid metabolism and amino acid metabolism according to TCGA LIHC dataset. **(B)** SMMC7721 cells were transfected with different dosages of Flag-PPP1R26. Western blot was done with the indicated antibodies (left). *PKM2/PKM1* mRNA levels were evaluated by RT-qPCR when Flag-PPP1R26 was expressed (right). **(C)** PPP1R26 was depleted by shRNA and Western blot was performed to evaluate the expression of PPP1R26, PKM1 and PKM2 (left). *PKM2/PKM1* mRNA levels were evaluated by RT-qPCR when PPP1R26 was depleted by shRNA in SMMC7721 cells (right). **(D)** The model for *PKM* splicing regulation [1]. **(E)** Huh7 and HepG2 cells were transfected with GFP or GFP-PPP1R26. Immunoprecipitation was performed with anti-PTBP1 antibody. The immunoprecipitated RNA was submitted to RT-qPCR to evaluate the enrichment of PKM fragment flanking exon 9 by PTBP1. The RNA enrichment was determined relative to the non-targeting IgG control. **(F)** Immunofluorescence staining was performed with anti-PPP1R26 and anti-PTBP1 antibodies. Nuclei were stained with DAPI (right). Immunoprecipitation was performed with anti-PPP1R26 antibodies on HepG2 cell lysates. Rad arrows point to the heavy chain of IgG (lower). Data information: In (B, C & E), data are presented as mean ± SD. Statistical significance was assessed using two-tailed t-tests (E) or one-way ANOVA with post hoc analysis LSD test (B & C). ***P* < 0.01, ****P* < 0.001, and *****P* < 0.0001.

**Fig. S3.**


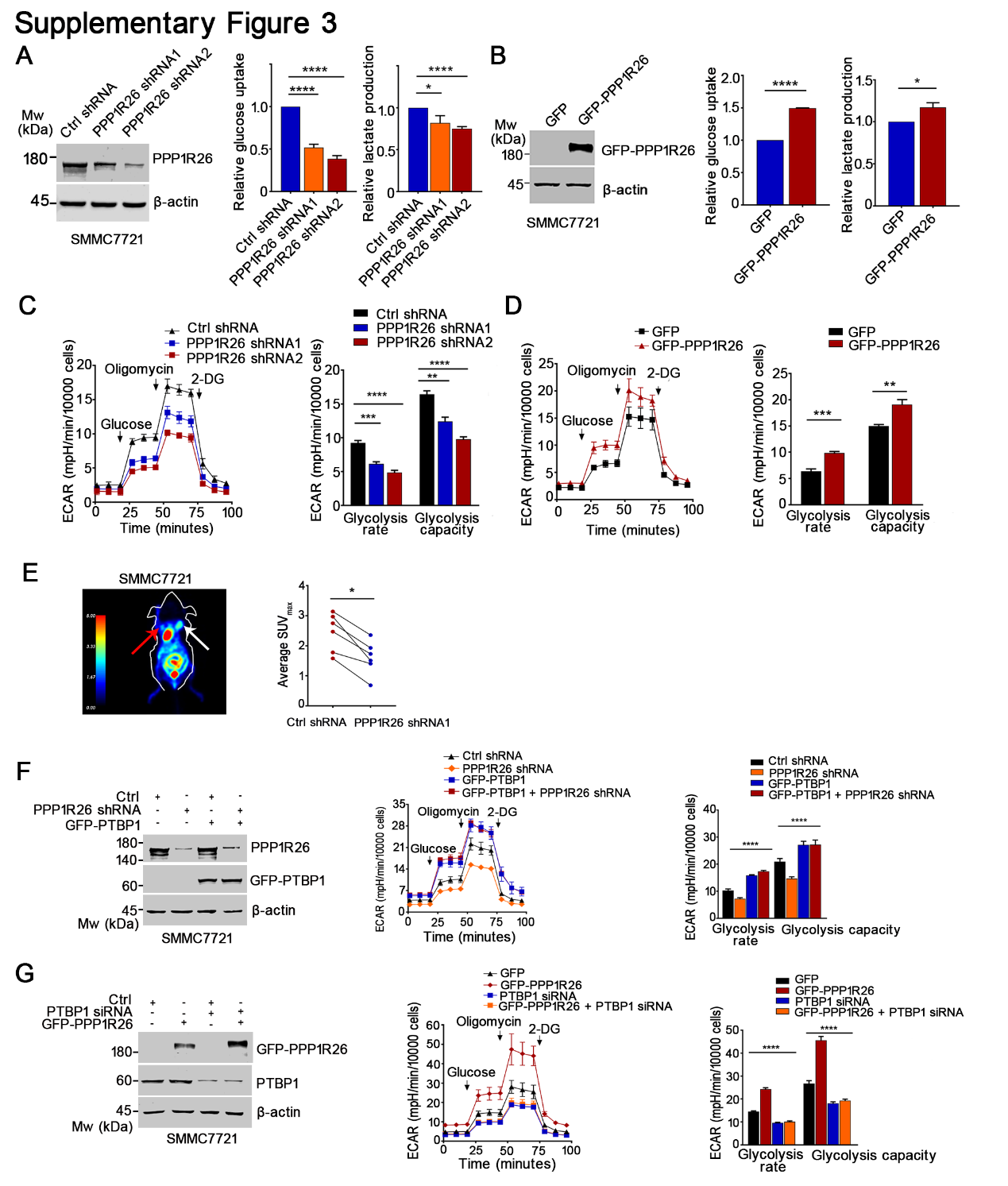


**Supplementary Fig. 3.** **PPP1R26 promotes glycolysis in SMMC7721 cells and *in vivo*.** **(A)** Expression of PPP1R26 was evaluated by Western blot in the SMMC7721 stably expressing shRNAs (left). β-actin was used as a loading control. Glucose uptake and lactate production in PPP1R26-depleted SMMC7721 were detected (middle & right). **(B)** GFP-PPP1R26 or GFP was transfected into SMMC7721. Expression of GFP-PPP1R26 was evaluated by Western blot (left). β-actin was used as a loading control. Glucose uptake and lactate production in SMMC7721 were detected (middle & right). **(C)** The extracellular acidification rate (ECAR) was evaluated in SMMC7721-PPP1R26 shRNA cells treated with glucose, oligomycin, and 2-deoxyglucose (2-DG), respectively (left). The histogram analysis of glycolysis rate (ECAR after glucose injection) and glycolysis capacity (ECAR after oligomycin injection) is shown (right). **(D)** SMMC7721 cells were transfected with GFP or GFP-PPP1R26. ECAR was evaluated as described. The histogram analysis showed glycolysis rate and glycolysis capacity (right). **(E)** SMMC7721-PPP1R26 shRNA cells were injected subcutaneously into the left and SMMC7721-Ctrl shRNA1 cells in the right flanks of BALB/C nude mice. PET/CT imaging was performed. Representative PET photographs of animals were shown (left). Glucose uptake in the tumor was evaluated by the average SUVmax (right). **(F)** Expression of PPP1R26 and GFP-PTBP1 were detected by Western blot in the indicated cells (left). β-actin was used as a loading control. ECAR was evaluated in PPP1R26-depleted SMMC7721 cells after being transfected with GFP-PTBP1 (middle). The histogram analysis showed the ECAR (right). **(G)** Expression of GFP-PPP1R26 and PTBP1 were detected by Western blot in the indicated cells (left). β-actin was used as a loading control. ECAR was evaluated in PTBP1-depleted SMMC7721 cells when transfected with GFP-PPP1R26 (middle). The histogram analysis showed the ECAR results (right). Data information: In (A-G), data are presented as mean ± SD. Statistical significance was assessed using two-tailed t-tests (B & D), one-way ANOVA with post hoc analysis LSD test (A, C, F & G) or Kruskal-Wallis test (E). **P* < 0.05, ***P* < 0.01, ****P* < 0.001 and *****P* < 0.0001.

**Fig. S4.**


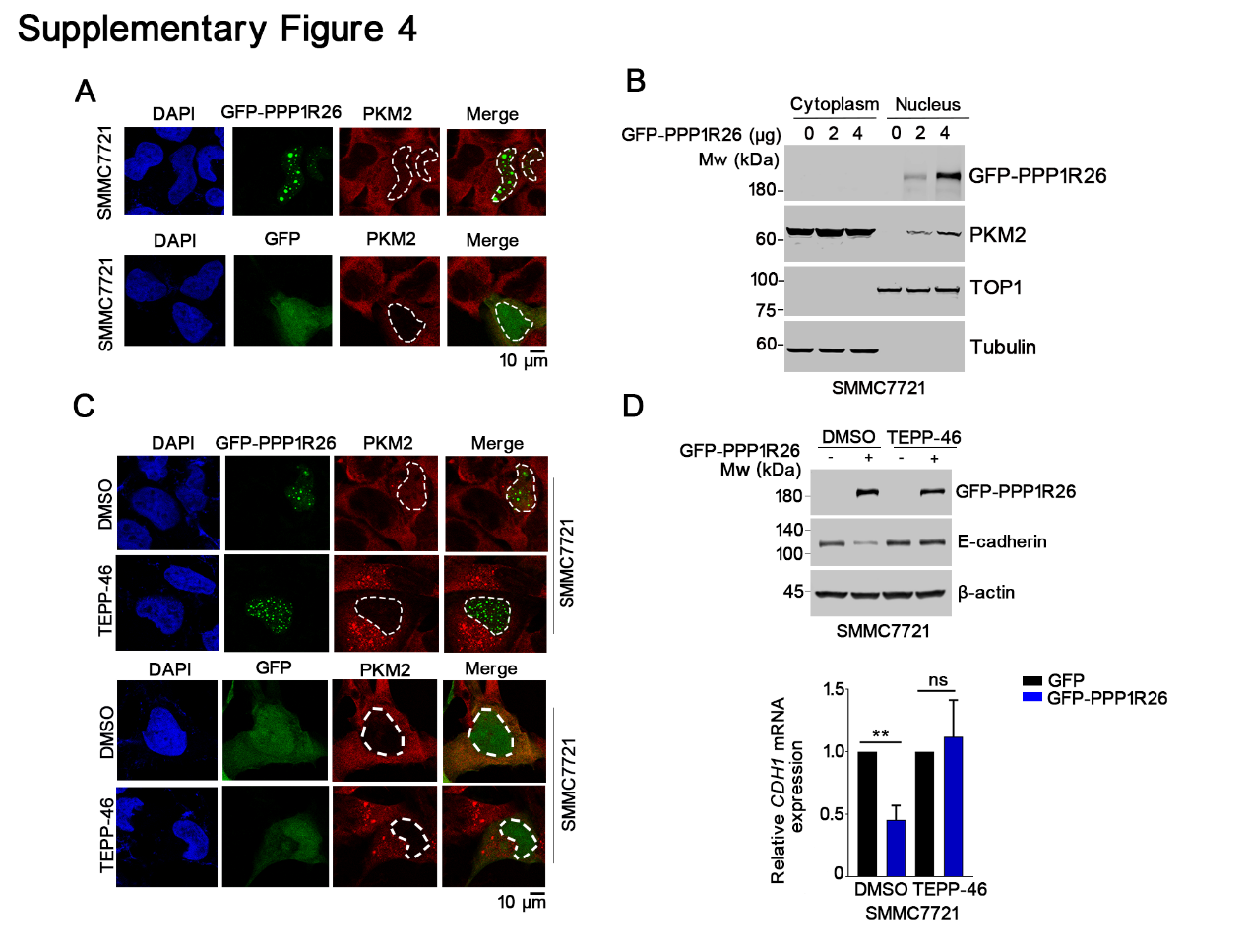


**Supplementary Fig. 4. Overexpressed PPP1R26 induces accumulation of nuclear PKM2 and promotes EMT in SMMC7721 cells dependent on nuclear PKM2. (A)** GFP-PPP1R26 (upper) or GFP (lower) was transfected into SMMC7721 cells. Cells were cultured under serum starvation and immunofluorescence staining was performed with anti-PKM2 antibody (red). DAPI was used to stain the nucleus. **(B)** SMMC7721 cells were transfected with GFP or GFP-PPP1R26 and cultured under serum starvation. Cytosolic and nuclear fractionations were prepared and the expression of PKM2 was determined by Western blot. TOP1 and Tubulin were used as the nuclear marker and cytoplasm marker, respectively. **(C)** Cells were treated with DMSO or TEPP-46 for 24h after GFP-PPP1R26 (upper) or GFP (lower) was transfected. Immunofluorescence staining was done with anti-PKM2 (red). DAPI stained nuclei. Immunofluorescence images were taken using a confocal microscope. **(D)** SMMC7721 cells were treated with DMSO or TEPP-46 for 24 h after GFP-PPP1R26 was transfected. Whole cell extracts were used to evaluate the expression of GFP-PPP1R26 and E-cadherin by Western blot (upper). Relative mRNA levels of *CDH1* after treatment with DMSO or TEPP-46 for 24 h in GFP-PPP1R26 or GFP expressing SMMC7721 cells (lower). Data information: In (D), data are presented as mean ± SD. Statistical significance was assessed using two-tailed t-tests (D). ***P* < 0.01.

**Fig. S5.**


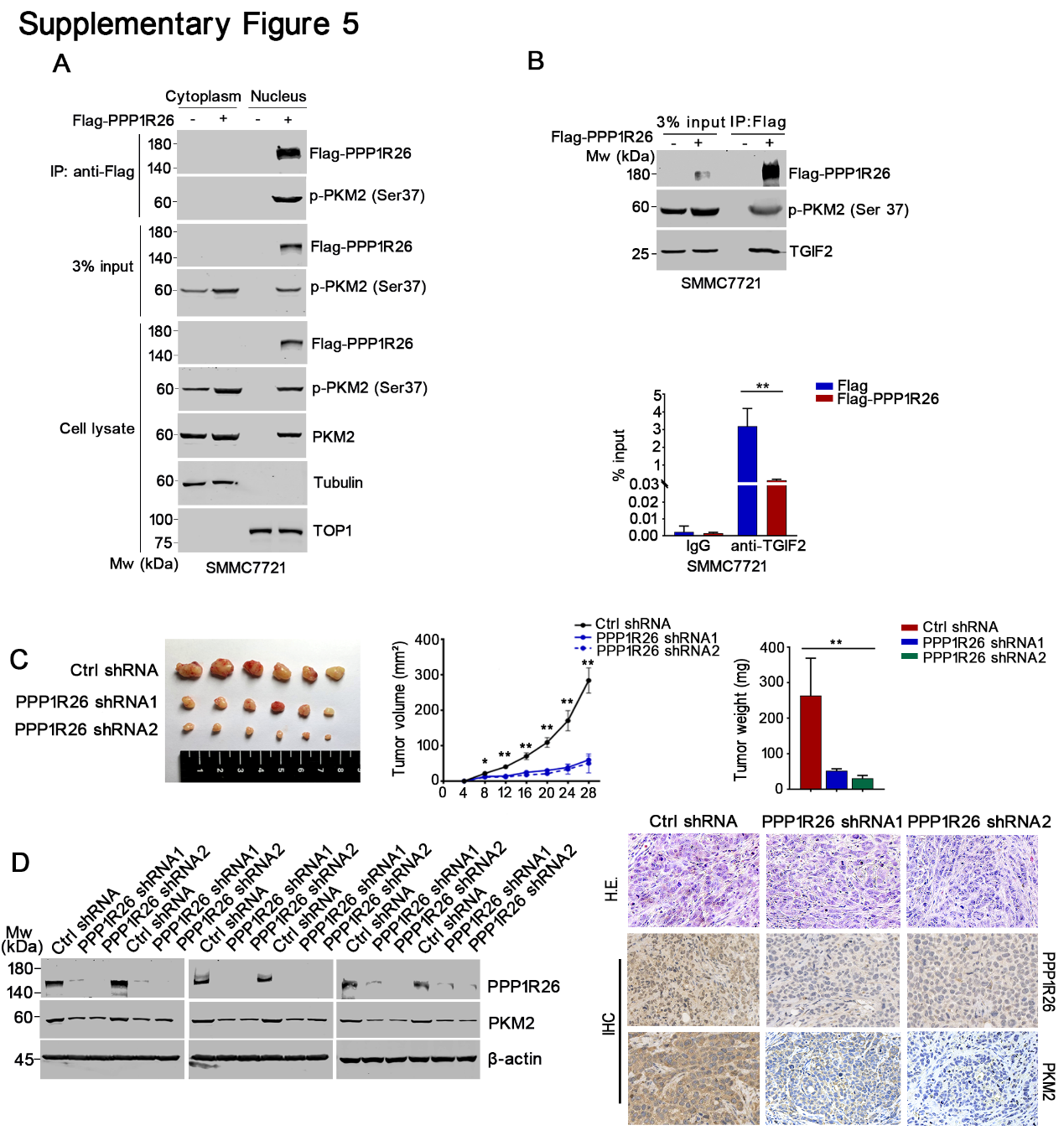


**Supplementary Fig. 5. PPP1R26 promotes EMT in SMMC7721 cells by the formation of the PPP1R26-pSer37 PKM2-TGIF2 complex. (A)** SMMC7721 cells were transfected with Flag or Flag-PPP1R26 and cultured under serum starvation. Immunoprecipitation was performed with an anti-Flag antibody on the cellular fractionation. The Flag-PPP1R26 and p-Ser37-PKM2 in the precipitates were determined by Western blot. **(B)** SMMC7721 cells were transfected with Flag or Flag-PPP1R26 and cultured under serum starvation. Immunoprecipitation was performed with an anti-Flag antibody. The Flag-PPP1R26, p-Ser37-PKM2 and TGIF2 in the precipitates were determined by Western blot (upper). SMMC7721 cells were transfected with Flag or Flag-PPP1R26, and ChIP assays were performed with IgG and anti-TGIF2 antibodies followed by qPCR (lower). **(C)** SMMC7721-PPP1R26-shRNA cells were subcutaneously implanted into nude mice. Tumors were dissected at the end of the experiment (left). Tumor volume and weight were evaluated as indicated (middle & right). **(D)** Proteins extracted from frozen xenografted tumor tissues were subjected to Western blotting probed with anti-PPP1R26 and anti-PKM2 antibodies (left). β-actin was used as a loading control. IHC was performed with anti-PPP1R26 and anti-PKM2 antibodies on the paraffin-embedded tumor tissues of xenografts (right). Data information: In (B & C), data are presented as mean ± SD. Statistical significance was assessed using two-tailed t-tests (B) or one-way ANOVA with post hoc analysis Kruskal-Wallis test (C). **P* < 0.05 and ***P* < 0.01.

**Fig. S6.**

**
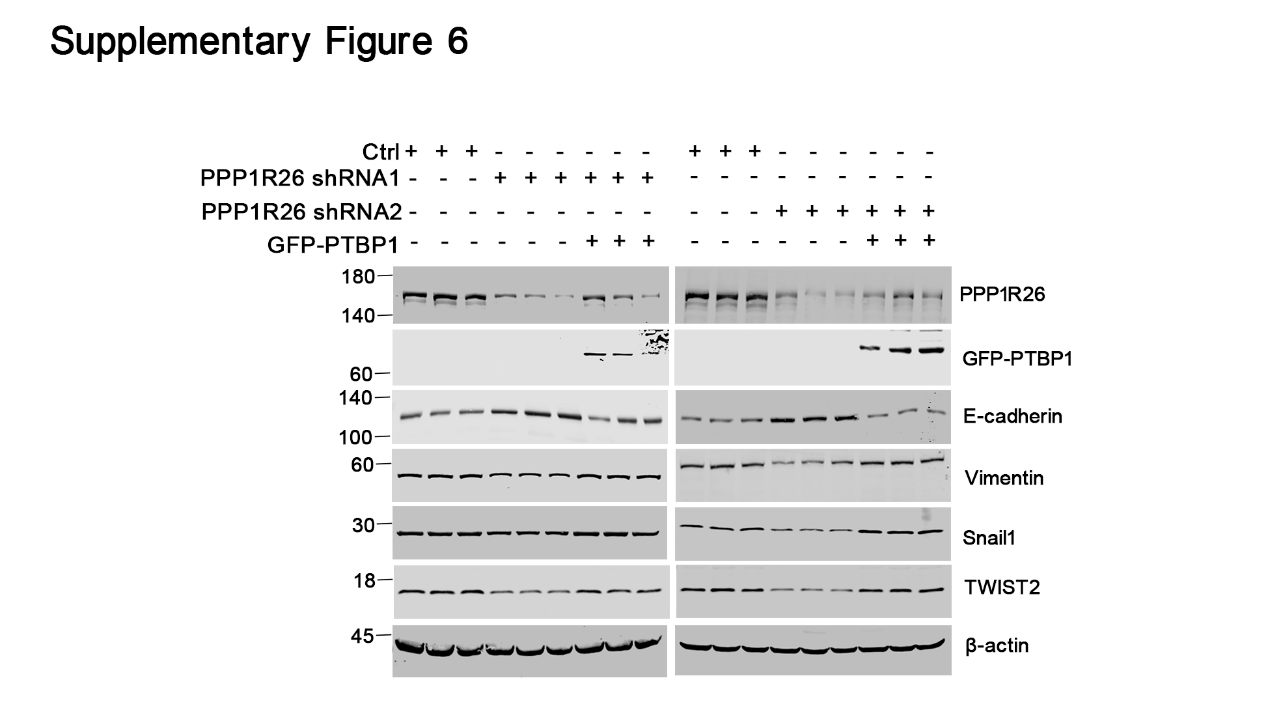
**

**Supplementary Fig. 6.** Proteins extracted from frozen xenografted tumor tissues (Huh7-PPP1R26-shRNA1, n = 6; Huh7-PPP1R26-shRNA-2, n = 6) were subjected to Western blotting probed with anti-PPP1R26, anti-GFP, anti-E-cadherin, anti-Vimentin, anti-Snail1 and anti-TWIST2 antibodies. β-actin was used as a loading control.

**Supplementary Tables 1-5.**

**Supplementary Table 1.** Reagents

| **Reagent** | **Company** | **Cat. No.** |
| --- | --- | --- |
| DMSO | Sigma-Aldrich | D2650 |
| TEPP-46 | Cayman Chemical | 13942 |
| Seahorse XF Glycolysis Stress Test Kit | Agilent Technologies | 103030-100 |
| Seahorse XFe24 FluxPak mini | Agilent Technologies | 102342-100 |
| XF DMEM Base Medium, pH 7.4 | Agilent Technologies | 103575-100 |
| XF 200mM Glutamine Solution | Agilent Technologies | 103579-100 |
| CellTiter 96 AQueous One Solution Cell Proliferation Assay system | Promega | G3582 |
| Amplex-Red Glucose assay kit | Thermo Fisher | A22189 |
| L-Lactate Assay Kit | Abcam | Ab65331L |
| DMEM | Thermo Fisher Gibco | 11995040 |
| DMEM (high glucose, no pyruvate)  DMEM (no glucose, no pyruvate)  RPMI 1640  Fetal bovine serum  Lipofectamine 2000 | Thermo Fisher Gibco  Thermo Fisher Gibco  Thermo Fisher Gibco  Thermo Fisher Gibco  Invitrogen | 11965084  11966025  11875119  16140071  12566014 |

**Supplementary Table 2.** shRNA and siRNA sequence

| **Gene** | **Sequence** |
| --- | --- |
| Control shRNA | 5’-TTCTCCGAACGTGTCACGTTT-3’  5’-ACGTGACACGTTCGGAGAATT-3’ |
| PPP1R26 shRNA-1  PPP1R26 shRNA-2  Control siRNA  PTBP1 siRNA-1  PTBP1 siRNA-2 | 5’-GAGCAGGAAATCCGGACATTT-3’  5’-ATGTCCGGATTTCCTGCTCTT-3’  5’-CAGGGCGGAAATAGAACAGTT-3’  5’-CTGTTCTATTTCCGCCCTGTT-3’  5’-UUCUCCGAACGUGUCACGUTT-3’  5’-ACGUGACACGUUCGGAGAATT-3’  5’-GCCUCAACGUCAAGUACAATT-3’  5’-UUGUACUUGACGUUGAGGCTT-3’  5’-GCACAGUGUUGAAGAUCAUTT-3’  5’-AUGAUCUUCAACACUGUGCTT-3’ |
| Control siRNA | 5’-CUUACGCUGAGUACUUCGA-3’  5’-UCGAAGUACUCAGCGUAAG-3’ |
| PKM2 siRNA | 5’-CCAUAAUCGUCCUCACCAA-3’  3’-UUGGUGAGGACGAUUAUGG-3’ |

**Supplementary Table 3.** Antibodies

| **Antibody** | **Company** | **Cat. No.** |
| --- | --- | --- |
| PKM1 | Cell Signaling Technology | 7067S |
| PKM2 | Cell Signaling Technology | 4053S |
| PTBP1 | Abcam | ab133734 |
| E-cadherin | Abcam | ab40772 |
| Vimentin | Abclonal | A19607 |
| Snail1 | Abclonal | A5243 |
| TWIST2 | Abclonal | A5599 |
| PKM2 (phospho-Ser37) | Signalway Antibody | 11456-2 |
| TGIF2 | Abcam | ab155948 |
| TOP1 | Abclonal | A12409 |
| Tubulin | Abclonal | AC012 |
| Flag | Sigma-Aldrich | F3165 |
| Flag | TransGen Biotech | HT201 |
| GFP | TransGen Biotech | HT801 |
| β-actin  IRDye® 680RD Goat anti-Rabbit IgG (H+L)  RDye® 800RD Goat anti-Mouse IgG (H+L) | Abclonal  LI-COR Biosciences  LI-COR Biosciences | AC026  926-68071  926-32210 |

**Supplementary Table 4.** Primer used for real-time PCR

| **Gene** | **Primer sequences** |
| --- | --- |
| *PKM1* | F: 5’-GGAGAAACAGCCAAAGGGGA-3’  R: 5’-TTGAGGCTCGCACAAGTTCT-3’ |
| *PKM2* | F:5’- AAGAACTTGTGCGAGCCTCA-3’  R: 5’-GACGAGCTGTCTGGGGATTC-3’ |
| *CDH1* | F: 5’-ATTTTTCCCTCGACACCCGAT-3’  R: 5’-TCCCAGGCGTAGACCAAGA-3’ |
| *SNAIL1* | F: 5’-GGTTCTTCTGCGCTACTGCT-3’ |
|  | R: 5’-TAGGGCTGCTGGAAGGTAAA-3’ |
| *TWIST2* | F: 5’-AGGAGCTGGACAGACAGCAG-3’ |
|  | R: 5’- TCAATGTACCTGGATGCGAG-3’ |
| *PTBP1* | F: 5’-GAAGGTGGTTATCGCAATCAGT-3’  R: 5’-TGCCAGATATAAGGACTCCGTT-3’ |
| *PKM* (for RIP)  *β-actin* | F: 5’-CTGTGCTACTCAGATGCTGGA-3’  R: 5’-CTGTGGAGTGACTTGAGGCT-3’  F: 5’-ATCGTCCACCGCAAATGCTTCTA-3’ |
|  | R: 5’-AGCCATGCCAATCTCATCTTGTT-3’ |
| *CDH1* promoter | F: 5’-GCTTGGGTGAAAGAGTGAGC-3’ |
|  | R: 5’-TAGGTGGGTTATGGGACCTG-3’ |

**Supplementary Table 5.** ssGSEA scores of 374 HCC samples obtained from TCGA based on the expression of metabolic related genes

| ID | Amino acid metabolism | Fatty acid metabolism | Glucose metabolism |
| --- | --- | --- | --- |
| TCGA-DD-AAEK-01A-11R-A41C-07 | 3.164455 | 2.950072 | 2.983748 |
| TCGA-DD-A4ND-01A-11R-A266-07 | 3.197749 | 2.971152 | 3.027795 |
| TCGA-2Y-A9H7-01A-11R-A39D-07 | 3.169274 | 2.820001 | 2.933085 |
| TCGA-RC-A7S9-01A-11R-A33R-07 | 3.19121 | 2.795434 | 2.950125 |
| TCGA-DD-AADI-01A-11R-A41C-07 | 3.088812 | 2.779223 | 2.909105 |
| TCGA-DD-A4NA-01A-11R-A266-07 | 2.863823 | 2.523009 | 2.904673 |
| TCGA-BC-A10T-01A-11R-A131-07 | 3.255355 | 2.970646 | 3.015683 |
| TCGA-RC-A7SH-01A-11R-A38B-07 | 3.068127 | 2.662917 | 2.897116 |
| TCGA-WJ-A86L-01A-12R-A39D-07 | 3.092778 | 2.800932 | 2.905628 |
| TCGA-DD-AA3A-01A-11R-A37K-07 | 2.937022 | 2.546578 | 2.929929 |
| TCGA-CC-A7IE-01A-21R-A38B-07 | 3.14466 | 2.701256 | 3.012431 |
| TCGA-DD-AAVS-01A-11R-A41C-07 | 3.186623 | 2.85338 | 2.943197 |
| TCGA-DD-AAE7-01A-11R-A41C-07 | 3.206899 | 2.958279 | 2.978906 |
| TCGA-BC-A3KG-01A-11R-A213-07 | 3.073877 | 2.809634 | 3.057164 |
| TCGA-DD-A3A2-01A-11R-A213-07 | 3.18818 | 2.965866 | 3.017612 |
| TCGA-DD-AAVR-01A-11R-A41C-07 | 3.230895 | 2.938796 | 2.957429 |
| TCGA-CC-A7II-01A-11R-A33J-07 | 2.941531 | 2.270223 | 2.927933 |
| TCGA-DD-AACN-01A-11R-A41C-07 | 3.168444 | 2.861192 | 2.96767 |
| TCGA-2Y-A9GX-01A-11R-A38B-07 | 3.212731 | 2.982575 | 3.006573 |
| TCGA-CC-A3MA-01A-11R-A213-07 | 2.997563 | 2.677839 | 2.963488 |
| TCGA-DD-AACQ-01A-11R-A41C-07 | 3.141169 | 2.870161 | 2.94642 |
| TCGA-RC-A7SF-01A-11R-A352-07 | 3.160083 | 2.904395 | 2.923962 |
| TCGA-DD-AADW-01A-11R-A39D-07 | 3.09134 | 2.769257 | 3.020236 |
| TCGA-G3-A3CH-01A-11R-A22L-07 | 3.224344 | 2.955215 | 2.977487 |
| TCGA-DD-A1EI-01A-11R-A131-07 | 3.17214 | 2.934634 | 2.979132 |
| TCGA-ZP-A9CZ-01A-11R-A38B-07 | 3.241858 | 2.949108 | 3.013532 |
| TCGA-KR-A7K0-01A-12R-A33R-07 | 3.201994 | 2.906193 | 2.948289 |
| TCGA-BC-A10Y-01A-11R-A131-07 | 3.201158 | 2.927509 | 2.979744 |
| TCGA-ED-A8O5-01A-11R-A36F-07 | 3.163543 | 2.753095 | 2.970074 |
| TCGA-CC-A9FS-01A-11R-A37K-07 | 3.237129 | 2.893063 | 2.898901 |
| TCGA-UB-A7MB-01A-11R-A33R-07 | 3.188512 | 2.885542 | 3.046218 |
| TCGA-ED-A627-01A-12R-A311-07 | 3.211439 | 3.006127 | 3.013932 |
| TCGA-XR-A8TE-01A-11R-A36F-07 | 3.100006 | 2.845266 | 2.946474 |
| TCGA-DD-AACF-01A-11R-A41C-07 | 3.196104 | 2.80726 | 2.948254 |
| TCGA-K7-A5RF-01A-11R-A28V-07 | 3.270223 | 3.045443 | 2.987601 |
| TCGA-ZP-A9D1-01A-11R-A38B-07 | 3.162473 | 2.91415 | 2.986612 |
| TCGA-DD-AADB-01A-11R-A41C-07 | 3.136564 | 2.779743 | 3.012194 |
| TCGA-DD-AAVZ-01A-11R-A41C-07 | 3.213491 | 2.926099 | 2.970906 |
| TCGA-BC-A216-01A-11R-A155-07 | 3.147029 | 2.856031 | 2.999707 |
| TCGA-DD-A1EF-01A-11R-A131-07 | 3.110184 | 2.787668 | 2.984382 |
| TCGA-DD-AADL-01A-11R-A41C-07 | 3.209634 | 2.956656 | 3.006109 |
| TCGA-KR-A7K8-01A-11R-A33J-07 | 3.131408 | 2.882358 | 3.033461 |
| TCGA-ED-A7PX-01A-51R-A352-07 | 2.977582 | 2.669089 | 2.949667 |
| TCGA-DD-AADK-01A-11R-A41C-07 | 3.186226 | 2.955562 | 2.937457 |
| TCGA-BW-A5NO-01A-11R-A27V-07 | 3.217882 | 2.938599 | 2.963054 |
| TCGA-DD-A39W-01A-11R-A213-07 | 3.098109 | 2.878687 | 2.943143 |
| TCGA-GJ-A9DB-01A-11R-A37K-07 | 3.219034 | 3.02215 | 3.006476 |
| TCGA-DD-A1EA-01A-11R-A131-07 | 3.120189 | 2.822328 | 3.005518 |
| TCGA-UB-A7MD-01A-12R-A352-07 | 3.212985 | 2.932728 | 3.000565 |
| TCGA-ES-A2HS-01A-11R-A180-07 | 3.213146 | 2.904763 | 2.865295 |
| TCGA-G3-A3CJ-01A-11R-A213-07 | 3.136695 | 2.822862 | 2.939167 |
| TCGA-FV-A3R2-01A-11R-A22L-07 | 3.193087 | 2.880065 | 2.957733 |
| TCGA-BW-A5NQ-01A-11R-A27V-07 | 3.157528 | 2.778154 | 3.004108 |
| TCGA-2Y-A9GZ-01A-11R-A39D-07 | 3.225163 | 2.939206 | 2.982038 |
| TCGA-4R-AA8I-01A-11R-A38B-07 | 3.265396 | 2.905584 | 2.98125 |
| TCGA-DD-A4NG-01A-11R-A27V-07 | 3.115736 | 2.883446 | 3.007409 |
| TCGA-RC-A6M5-01A-11R-A32O-07 | 3.239748 | 2.907368 | 2.95982 |
| TCGA-2Y-A9H0-01A-11R-A38B-07 | 3.185758 | 2.884483 | 3.014963 |
| TCGA-DD-A11B-01A-11R-A131-07 | 3.238188 | 2.929053 | 2.980993 |
| TCGA-G3-A25V-01A-11R-A16W-07 | 3.246641 | 3.024517 | 2.994357 |
| TCGA-DD-AADQ-01A-11R-A41C-07 | 3.200997 | 2.805054 | 2.920426 |
| TCGA-CC-A8HS-01A-11R-A36F-07 | 3.071862 | 2.675432 | 2.993025 |
| TCGA-DD-A1EL-01A-11R-A155-07 | 3.155981 | 2.797597 | 2.994798 |
| TCGA-WQ-AB4B-01A-11R-A41C-07 | 3.1916 | 2.919572 | 2.929212 |
| TCGA-5R-AA1C-01A-11R-A41C-07 | 3.130585 | 2.882512 | 2.920105 |
| TCGA-ED-A8O6-01A-11R-A36F-07 | 3.132525 | 2.82186 | 2.948103 |
| TCGA-DD-AADD-01A-11R-A41C-07 | 3.13932 | 2.82212 | 3.008146 |
| TCGA-BC-A10S-01A-22R-A131-07 | 3.215998 | 2.966794 | 2.957346 |
| TCGA-CC-A9FV-01A-11R-A37K-07 | 2.878575 | 2.499709 | 2.855248 |
| TCGA-DD-AAEB-01A-11R-A41C-07 | 3.157597 | 2.839203 | 2.856645 |
| TCGA-WX-AA44-01A-11R-A39D-07 | 3.127331 | 2.886849 | 2.989629 |
| TCGA-PD-A5DF-01A-11R-A27V-07 | 3.148106 | 2.910917 | 3.022441 |
| TCGA-YA-A8S7-01A-11R-A37K-07 | 3.055503 | 2.722096 | 2.972449 |
| TCGA-5R-AA1D-01A-11R-A38B-07 | 3.211204 | 2.903132 | 2.963769 |
| TCGA-DD-AACH-01A-11R-A41C-07 | 3.148819 | 2.835106 | 2.994781 |
| TCGA-CC-A8HV-01A-11R-A36F-07 | 3.027719 | 2.705431 | 2.955894 |
| TCGA-BW-A5NP-01A-11R-A27V-07 | 3.115227 | 2.687241 | 3.028899 |
| TCGA-G3-AAV0-01A-11R-A37K-07 | 3.165103 | 2.838615 | 2.932011 |
| TCGA-DD-AAED-01A-12R-A41C-07 | 3.09655 | 2.759589 | 2.884171 |
| TCGA-ZS-A9CG-01A-11R-A37K-07 | 3.192031 | 2.929668 | 2.917962 |
| TCGA-K7-A6G5-01A-11R-A311-07 | 3.177523 | 3.004467 | 2.952749 |
| TCGA-DD-AADS-01A-11R-A41C-07 | 3.222418 | 2.847023 | 2.899183 |
| TCGA-DD-A3A3-01A-11R-A22L-07 | 3.168277 | 2.865172 | 2.890291 |
| TCGA-2Y-A9H8-01A-11R-A39D-07 | 3.18795 | 2.882568 | 3.034525 |
| TCGA-G3-A7M6-01A-11R-A33R-07 | 3.052262 | 2.700952 | 2.947452 |
| TCGA-FV-A2QQ-01A-11R-A22L-07 | 3.222559 | 2.930385 | 2.985999 |
| TCGA-ZP-A9D4-01A-11R-A37K-07 | 3.158471 | 2.84987 | 2.951538 |
| TCGA-LG-A9QD-01A-11R-A38B-07 | 3.22836 | 2.953878 | 2.953081 |
| TCGA-RC-A6M4-01A-11R-A32O-07 | 3.162224 | 2.881759 | 2.979366 |
| TCGA-ED-A7XP-01A-11R-A352-07 | 3.193114 | 2.845529 | 2.96254 |
| TCGA-2Y-A9HA-01A-11R-A39D-07 | 3.206452 | 2.925316 | 3.016764 |
| TCGA-DD-AAE2-01A-11R-A41C-07 | 3.233311 | 2.952843 | 3.05408 |
| TCGA-DD-AADF-01A-11R-A41C-07 | 3.190645 | 2.793889 | 2.992923 |
| TCGA-DD-AACW-01A-11R-A41C-07 | 3.162614 | 2.827872 | 2.985343 |
| TCGA-MI-A75I-01A-11R-A32O-07 | 3.196116 | 2.936837 | 3.000703 |
| TCGA-FV-A3I0-01A-11R-A22L-07 | 2.941476 | 2.506008 | 2.918457 |
| TCGA-2Y-A9H1-01A-11R-A38B-07 | 3.18237 | 2.863782 | 2.86826 |
| TCGA-CC-A8HU-01A-11R-A36F-07 | 3.176063 | 2.833946 | 3.081258 |
| TCGA-DD-AAD2-01A-11R-A41C-07 | 3.242553 | 3.023253 | 3.010247 |
| TCGA-BC-A10R-01A-11R-A131-07 | 3.208359 | 2.893683 | 2.930423 |
| TCGA-G3-A5SL-01A-11R-A27V-07 | 3.167204 | 2.953176 | 2.990273 |
| TCGA-CC-A1HT-01A-11R-A131-07 | 2.935937 | 2.569106 | 2.953701 |
| TCGA-2Y-A9GV-01A-11R-A38B-07 | 3.197059 | 2.910857 | 2.999288 |
| TCGA-ED-A7XO-01A-11R-A352-07 | 3.2471 | 2.971758 | 2.99053 |
| TCGA-CC-A5UC-01A-11R-A28V-07 | 3.203078 | 2.887079 | 3.021771 |
| TCGA-UB-A7ME-01A-11R-A33J-07 | 3.134245 | 2.909968 | 2.936714 |
| TCGA-G3-A7M9-01A-23R-A352-07 | 3.131848 | 2.678708 | 3.120859 |
| TCGA-DD-AACJ-01A-11R-A41C-07 | 3.168702 | 2.871026 | 2.939238 |
| TCGA-DD-A4NK-01A-11R-A28V-07 | 3.206319 | 2.942587 | 3.001822 |
| TCGA-DD-A3A5-01A-11R-A22L-07 | 3.23409 | 2.913749 | 2.937074 |
| TCGA-G3-A7M7-01A-12R-A352-07 | 3.232683 | 2.934944 | 2.96622 |
| TCGA-G3-AAUZ-01A-11R-A38B-07 | 3.230695 | 2.97067 | 2.904229 |
| TCGA-RC-A7SK-01A-11R-A352-07 | 3.207312 | 2.916585 | 2.942794 |
| TCGA-DD-AACI-01A-11R-A41C-07 | 3.117563 | 2.882742 | 2.91922 |
| TCGA-2V-A95S-01A-11R-A37K-07 | 3.047189 | 2.681326 | 2.912422 |
| TCGA-DD-A4NH-01A-11R-A27V-07 | 3.073278 | 2.724677 | 3.006404 |
| TCGA-WX-AA46-01A-11R-A39D-07 | 3.220861 | 3.007412 | 2.979345 |
| TCGA-DD-A4NQ-01A-21R-A28V-07 | 3.152798 | 2.875141 | 3.082479 |
| TCGA-G3-A5SM-01A-12R-A28V-07 | 3.221389 | 3.003783 | 2.97385 |
| TCGA-MI-A75E-01A-11R-A32O-07 | 3.215777 | 2.978056 | 2.981989 |
| TCGA-DD-A11D-01A-11R-A131-07 | 3.170658 | 2.99319 | 3.05566 |
| TCGA-CC-5262-01A-01R-A131-07 | 3.202631 | 2.950671 | 2.972306 |
| TCGA-2Y-A9GY-01A-11R-A38B-07 | 3.133857 | 2.713415 | 2.984646 |
| TCGA-ES-A2HT-01A-12R-A180-07 | 3.257091 | 3.032014 | 2.970346 |
| TCGA-DD-AAW0-01A-11R-A41C-07 | 3.241662 | 3.011169 | 3.034507 |
| TCGA-G3-A3CI-01A-11R-A213-07 | 3.203289 | 3.024727 | 2.998119 |
| TCGA-DD-AAVV-01A-11R-A41C-07 | 3.119152 | 2.885142 | 2.978094 |
| TCGA-DD-A39Y-01A-11R-A213-07 | 3.136163 | 2.795649 | 3.023879 |
| TCGA-DD-A1EB-01A-11R-A131-07 | 3.121785 | 2.868895 | 3.035982 |
| TCGA-DD-AAD1-01A-11R-A41C-07 | 3.109055 | 2.866875 | 2.940613 |
| TCGA-G3-A3CK-01A-11R-A213-07 | 3.175504 | 2.835463 | 2.981129 |
| TCGA-DD-AACA-02A-11R-A41C-07 | 3.13889 | 2.863873 | 2.952593 |
| TCGA-5C-A9VH-01A-11R-A37K-07 | 3.154911 | 2.862076 | 2.89612 |
| TCGA-FV-A23B-01A-11R-A16W-07 | 3.166931 | 2.783155 | 2.971527 |
| TCGA-DD-AADR-01A-11R-A41C-07 | 3.208886 | 2.923694 | 2.930165 |
| TCGA-DD-A4NO-01A-11R-A28V-07 | 3.213677 | 2.95102 | 2.947232 |
| TCGA-BC-A10W-01A-11R-A131-07 | 3.07876 | 2.828934 | 3.073305 |
| TCGA-LG-A9QC-01A-11R-A37K-07 | 3.070459 | 2.876323 | 2.975519 |
| TCGA-DD-A11C-01A-11R-A131-07 | 3.197486 | 2.903149 | 3.00957 |
| TCGA-DD-AACV-01A-11R-A41C-07 | 3.194681 | 2.874622 | 3.031246 |
| TCGA-CC-5264-01A-01R-A131-07 | 3.055783 | 2.841357 | 3.010572 |
| TCGA-DD-AACL-01A-11R-A41C-07 | 3.151812 | 2.803209 | 3.027479 |
| TCGA-DD-A116-01A-11R-A131-07 | 3.194878 | 2.964849 | 2.940397 |
| TCGA-CC-A7IG-01A-11R-A33J-07 | 3.079267 | 2.727591 | 2.970207 |
| TCGA-DD-AAE9-01A-11R-A41C-07 | 3.132111 | 2.777379 | 2.856943 |
| TCGA-DD-AAVP-01A-11R-A41C-07 | 3.16029 | 2.935264 | 3.006901 |
| TCGA-G3-AAV5-01A-11R-A37K-07 | 3.14254 | 2.800555 | 2.92383 |
| TCGA-ZS-A9CF-02A-11R-A38B-07 | 3.192903 | 2.867534 | 2.960926 |
| TCGA-NI-A8LF-01A-11R-A36F-07 | 3.188207 | 2.91839 | 2.946824 |
| TCGA-DD-A39Z-01A-11R-A213-07 | 3.126662 | 2.811274 | 2.949203 |
| TCGA-ZS-A9CF-01A-11R-A38B-07 | 3.188806 | 2.93816 | 2.967347 |
| TCGA-EP-A3RK-01A-11R-A22L-07 | 3.144997 | 2.918525 | 2.975835 |
| TCGA-FV-A496-01A-11R-A266-07 | 3.148213 | 2.870344 | 3.042682 |
| TCGA-CC-A3MB-01A-11R-A213-07 | 3.152634 | 2.895049 | 3.026111 |
| TCGA-CC-A7IF-01A-11R-A33J-07 | 3.219563 | 2.868088 | 2.900853 |
| TCGA-ZS-A9CD-01A-11R-A37K-07 | 3.186089 | 2.93658 | 2.966053 |
| TCGA-G3-A25S-01A-11R-A16W-07 | 3.182645 | 2.840445 | 2.948154 |
| TCGA-BC-A217-01A-11R-A155-07 | 3.178511 | 2.893443 | 2.962103 |
| TCGA-DD-AAW2-01A-11R-A41C-07 | 3.180808 | 2.880847 | 2.926622 |
| TCGA-ED-A97K-01A-21R-A38B-07 | 3.07652 | 2.84127 | 3.015806 |
| TCGA-DD-AAVW-01A-11R-A41C-07 | 3.189392 | 2.96778 | 2.943241 |
| TCGA-DD-A73F-01A-11R-A32O-07 | 3.247842 | 2.954727 | 3.029416 |
| TCGA-DD-A4NN-01A-11R-A28V-07 | 3.068757 | 2.717639 | 2.955894 |
| TCGA-DD-AAEI-01A-11R-A41C-07 | 3.183935 | 2.914342 | 3.030462 |
| TCGA-UB-AA0V-01A-11R-A38B-07 | 3.224285 | 2.997888 | 2.982613 |
| TCGA-DD-AADO-01A-11R-A41C-07 | 3.137228 | 2.806731 | 2.953471 |
| TCGA-DD-AAVY-01A-11R-A41C-07 | 3.221003 | 2.769882 | 2.973227 |
| TCGA-FV-A3R3-01A-11R-A22L-07 | 3.213858 | 2.918711 | 2.981438 |
| TCGA-DD-A4NS-01A-11R-A311-07 | 3.197781 | 2.989787 | 2.99113 |
| TCGA-MI-A75H-01A-11R-A32O-07 | 3.192943 | 2.868406 | 2.930664 |
| TCGA-UB-A7MC-01A-11R-A33R-07 | 3.125702 | 2.775432 | 2.987601 |
| TCGA-FV-A4ZQ-01A-11R-A266-07 | 3.15713 | 2.832051 | 2.970199 |
| TCGA-DD-AAEG-01A-11R-A39D-07 | 3.201966 | 2.97512 | 2.999866 |
| TCGA-DD-AAD5-01A-11R-A41C-07 | 3.035877 | 2.765841 | 2.969228 |
| TCGA-K7-A5RG-01A-11R-A28V-07 | 3.201282 | 2.86935 | 3.048412 |
| TCGA-XR-A8TD-01A-12R-A39D-07 | 3.198537 | 2.980056 | 3.089943 |
| TCGA-DD-AACY-01A-11R-A41C-07 | 3.216343 | 2.904571 | 2.96646 |
| TCGA-CC-A9FU-01A-11R-A37K-07 | 3.123057 | 2.634446 | 2.983387 |
| TCGA-KR-A7K7-01A-11R-A33J-07 | 3.133862 | 2.756923 | 2.938834 |
| TCGA-G3-A25Y-01A-11R-A16W-07 | 3.144839 | 2.85001 | 2.995264 |
| TCGA-K7-AAU7-01A-11R-A38B-07 | 3.090284 | 2.816528 | 2.987126 |
| TCGA-DD-AACA-02B-11R-A41C-07 | 3.112059 | 2.806095 | 2.930798 |
| TCGA-BC-A3KF-01A-11R-A213-07 | 3.191811 | 2.953363 | 2.975764 |
| TCGA-2Y-A9H2-01A-12R-A38B-07 | 3.12754 | 2.814773 | 2.974603 |
| TCGA-DD-AAW3-01A-11R-A41C-07 | 3.136821 | 2.844399 | 2.931731 |
| TCGA-ED-A82E-01A-11R-A352-07 | 2.879695 | 2.575033 | 2.818841 |
| TCGA-DD-A4NP-01A-11R-A28V-07 | 3.190827 | 2.923014 | 2.92557 |
| TCGA-G3-A6UC-01A-21R-A33J-07 | 3.188246 | 2.823923 | 2.963428 |
| TCGA-DD-AAW1-01A-11R-A41C-07 | 3.158144 | 2.863012 | 2.915426 |
| TCGA-DD-A118-01A-11R-A131-07 | 3.184653 | 2.790328 | 2.949261 |
| TCGA-DD-AADA-01A-11R-A41C-07 | 3.110957 | 2.774818 | 2.981794 |
| TCGA-ED-A66Y-01A-11R-A311-07 | 3.031648 | 2.669161 | 2.945914 |
| TCGA-DD-AAE3-01A-11R-A41C-07 | 3.242749 | 2.913216 | 2.97257 |
| TCGA-G3-AAV6-01A-21R-A37K-07 | 3.117844 | 2.678609 | 2.98645 |
| TCGA-DD-A4NE-01A-11R-A27V-07 | 3.131914 | 2.882701 | 3.026105 |
| TCGA-DD-AADV-01A-11R-A39D-07 | 3.236007 | 2.950461 | 2.98772 |
| TCGA-DD-AACA-01A-11R-A41C-07 | 3.14388 | 2.852566 | 2.942075 |
| TCGA-DD-AACX-01A-11R-A41C-07 | 3.122813 | 2.837426 | 3.009345 |
| TCGA-DD-AACC-01A-11R-A41C-07 | 3.169635 | 2.924626 | 3.013628 |
| TCGA-G3-A5SK-01A-11R-A27V-07 | 3.222875 | 2.870065 | 2.913577 |
| TCGA-CC-A3MC-01A-11R-A22L-07 | 3.192979 | 2.970552 | 3.060699 |
| TCGA-DD-A73A-01A-12R-A32O-07 | 3.200233 | 2.899451 | 2.968079 |
| TCGA-DD-AADG-01A-11R-A41C-07 | 3.152256 | 2.845366 | 2.97654 |
| TCGA-DD-A4NL-01A-11R-A28V-07 | 3.255338 | 3.015577 | 2.976087 |
| TCGA-BC-A10Q-01A-11R-A131-07 | 2.887823 | 2.700031 | 2.917313 |
| TCGA-2Y-A9GU-01A-11R-A38B-07 | 3.218494 | 2.847125 | 2.991069 |
| TCGA-ZP-A9CV-01A-11R-A38B-07 | 3.200873 | 3.030253 | 2.986602 |
| TCGA-DD-A113-01A-11R-A131-07 | 3.186372 | 2.877124 | 3.014795 |
| TCGA-2Y-A9GT-01A-11R-A38B-07 | 3.196544 | 2.96921 | 2.949298 |
| TCGA-DD-A39X-01A-11R-A213-07 | 3.211188 | 2.883124 | 2.925354 |
| TCGA-RC-A6M3-01A-11R-A32O-07 | 3.124138 | 2.652282 | 3.028967 |
| TCGA-DD-AACU-01A-11R-A41C-07 | 3.196484 | 3.004572 | 2.993364 |
| TCGA-WX-AA47-01A-11R-A39D-07 | 3.191459 | 2.682526 | 2.917937 |
| TCGA-DD-A3A4-01A-11R-A22L-07 | 3.220388 | 2.848348 | 2.965578 |
| TCGA-ED-A7PY-01A-11R-A33R-07 | 3.147793 | 2.839565 | 2.889871 |
| TCGA-EP-A2KC-01A-11R-A213-07 | 3.167164 | 2.926129 | 2.942885 |
| TCGA-BC-A10X-01A-11R-A131-07 | 3.249666 | 3.032181 | 3.033177 |
| TCGA-CC-A7IH-01A-11R-A33J-07 | 3.120986 | 2.833797 | 2.918946 |
| TCGA-FV-A495-01A-11R-A266-07 | 3.205022 | 2.98878 | 2.924246 |
| TCGA-DD-A1EH-01A-11R-A131-07 | 3.089794 | 2.697132 | 3.005879 |
| TCGA-DD-AAEH-01A-11R-A41C-07 | 3.250931 | 2.9563 | 2.961933 |
| TCGA-DD-AAEE-01A-11R-A41C-07 | 3.219485 | 2.902039 | 2.95464 |
| TCGA-ZP-A9D0-01A-11R-A37K-07 | 3.255219 | 2.880955 | 2.987836 |
| TCGA-DD-A1EG-01A-11R-A213-07 | 3.170254 | 2.885447 | 2.998889 |
| TCGA-2Y-A9H6-01A-11R-A39D-07 | 3.221337 | 2.944791 | 2.966829 |
| TCGA-2Y-A9GW-01A-11R-A38B-07 | 3.169426 | 2.954628 | 3.019411 |
| TCGA-DD-A73D-01A-12R-A32O-07 | 3.151465 | 2.786802 | 2.96084 |
| TCGA-DD-A115-01A-11R-A131-07 | 3.217259 | 2.975118 | 2.983768 |
| TCGA-XR-A8TF-01A-11R-A36F-07 | 3.201814 | 2.810739 | 2.986728 |
| TCGA-DD-A4NI-01A-11R-A27V-07 | 3.249141 | 2.950886 | 2.980641 |
| TCGA-G3-AAV3-01A-11R-A37K-07 | 3.163641 | 2.914085 | 2.943625 |
| TCGA-UB-A7MA-01A-11R-A33R-07 | 3.041909 | 2.68405 | 2.946773 |
| TCGA-UB-A7MF-01A-11R-A33J-07 | 3.201815 | 2.771468 | 3.015803 |
| TCGA-BC-A112-01A-11R-A131-07 | 3.033964 | 2.804934 | 3.002199 |
| TCGA-BC-A10U-01A-11R-A131-07 | 3.177085 | 2.87808 | 3.052164 |
| TCGA-BC-A5W4-01A-11R-A28V-07 | 3.120335 | 2.817675 | 2.918628 |
| TCGA-FV-A3I1-01A-11R-A22L-07 | 3.160067 | 2.964821 | 3.033204 |
| TCGA-CC-A123-01A-11R-A131-07 | 3.104136 | 2.769612 | 2.873251 |
| TCGA-WQ-A9G7-01A-11R-A37K-07 | 3.136082 | 2.824046 | 2.955994 |
| TCGA-ED-A66X-01A-11R-A311-07 | 3.151316 | 2.843203 | 2.99934 |
| TCGA-DD-A1EK-01A-11R-A213-07 | 3.199539 | 3.049808 | 3.063603 |
| TCGA-DD-AADM-01A-11R-A41C-07 | 3.219123 | 2.956261 | 2.945635 |
| TCGA-MR-A8JO-01A-12R-A36F-07 | 3.170617 | 2.766102 | 2.960057 |
| TCGA-QA-A7B7-01A-11R-A32O-07 | 3.165971 | 2.829779 | 3.05467 |
| TCGA-CC-A8HT-01A-11R-A36F-07 | 3.130591 | 2.844617 | 3.036489 |
| TCGA-FV-A4ZP-01A-12R-A266-07 | 2.984792 | 2.642394 | 2.880096 |
| TCGA-DD-A4NJ-01A-11R-A27V-07 | 3.162207 | 2.897783 | 3.046789 |
| TCGA-CC-5258-01A-01R-A131-07 | 3.183251 | 2.789477 | 3.074239 |
| TCGA-EP-A2KB-01A-11R-A180-07 | 3.180219 | 2.771737 | 2.946488 |
| TCGA-G3-A25X-01A-11R-A16W-07 | 3.090814 | 2.709397 | 2.958832 |
| TCGA-CC-A5UD-01A-11R-A28V-07 | 3.064551 | 2.696796 | 2.976368 |
| TCGA-DD-A119-01A-11R-A131-07 | 3.235585 | 2.948451 | 2.952082 |
| TCGA-DD-AACB-01A-11R-A41C-07 | 3.141594 | 2.75903 | 2.953866 |
| TCGA-DD-AAE4-01A-11R-A41C-07 | 3.241819 | 2.950948 | 2.936573 |
| TCGA-CC-A3M9-01A-11R-A213-07 | 2.826473 | 2.354308 | 3.00508 |
| TCGA-CC-A7IL-01A-11R-A33R-07 | 3.203318 | 2.875895 | 2.970896 |
| TCGA-BC-A10Z-01A-11R-A131-07 | 3.197154 | 2.992361 | 2.982581 |
| TCGA-UB-AA0U-01A-11R-A38B-07 | 3.16648 | 3.018511 | 3.055582 |
| TCGA-DD-A3A8-01A-11R-A22L-07 | 3.154536 | 2.90872 | 2.945269 |
| TCGA-ED-A4XI-01A-11R-A266-07 | 3.240802 | 3.044033 | 2.976965 |
| TCGA-HP-A5MZ-01A-21R-A27V-07 | 3.190818 | 2.98914 | 2.970631 |
| TCGA-DD-AAVU-01A-11R-A41C-07 | 3.181578 | 2.803639 | 2.881598 |
| TCGA-DD-AAD6-01A-11R-A41C-07 | 3.083132 | 2.825417 | 2.983252 |
| TCGA-EP-A26S-01A-11R-A16W-07 | 3.181089 | 2.894429 | 2.96398 |
| TCGA-DD-AAVX-01A-11R-A41C-07 | 3.19935 | 2.868239 | 2.970055 |
| TCGA-ED-A7PZ-01A-11R-A33R-07 | 3.182654 | 2.804835 | 3.023317 |
| TCGA-LG-A6GG-01A-11R-A311-07 | 3.166741 | 2.927033 | 2.960037 |
| TCGA-DD-AADC-01A-11R-A41C-07 | 3.121436 | 2.790944 | 2.970993 |
| TCGA-DD-A3A7-01A-11R-A22L-07 | 3.111512 | 2.758679 | 2.941281 |
| TCGA-DD-AACE-01A-11R-A41C-07 | 3.122617 | 2.870924 | 3.021427 |
| TCGA-2Y-A9H3-01A-11R-A38B-07 | 3.188062 | 2.799457 | 2.912738 |
| TCGA-DD-A4NB-01A-12R-A266-07 | 3.218872 | 2.866419 | 2.976128 |
| TCGA-G3-A5SI-01A-31R-A27V-07 | 3.166662 | 2.762946 | 2.95839 |
| TCGA-DD-AAE6-01A-11R-A41C-07 | 3.157792 | 2.83189 | 2.997348 |
| TCGA-DD-AACG-01A-11R-A41C-07 | 3.181392 | 2.846408 | 2.954735 |
| TCGA-5C-A9VG-01A-11R-A37K-07 | 3.214376 | 2.781625 | 3.014149 |
| TCGA-DD-A1EE-01A-11R-A131-07 | 3.124722 | 2.844866 | 3.019142 |
| TCGA-BC-A110-01A-11R-A131-07 | 3.234282 | 3.007017 | 3.028244 |
| TCGA-GJ-A3OU-01A-31R-A38B-07 | 3.119274 | 2.946236 | 3.042032 |
| TCGA-G3-A25U-01A-11R-A16W-07 | 3.15436 | 2.823398 | 2.983774 |
| TCGA-G3-A3CG-01A-11R-A213-07 | 3.188789 | 2.915507 | 2.998685 |
| TCGA-CC-5260-01A-01R-A131-07 | 2.851286 | 2.577737 | 2.967386 |
| TCGA-CC-A7IJ-01A-11R-A33R-07 | 2.897383 | 2.407756 | 2.945157 |
| TCGA-DD-AADU-01A-11R-A41C-07 | 3.099327 | 2.829629 | 2.899981 |
| TCGA-DD-AAE1-01A-11R-A41C-07 | 3.135623 | 2.779958 | 2.943116 |
| TCGA-2Y-A9HB-01A-11R-A39D-07 | 3.220885 | 2.996072 | 3.02106 |
| TCGA-BD-A2L6-01A-11R-A213-07 | 3.188198 | 2.940609 | 2.982194 |
| TCGA-DD-A3A9-01A-11R-A266-07 | 3.084014 | 2.74661 | 2.950474 |
| TCGA-KR-A7K2-01A-12R-A33R-07 | 3.203509 | 2.820266 | 2.889049 |
| TCGA-DD-A3A1-01A-11R-A213-07 | 3.192617 | 2.975056 | 2.948472 |
| TCGA-DD-A4NR-01A-11R-A311-07 | 3.172966 | 2.896626 | 3.044998 |
| TCGA-DD-AAC9-01A-11R-A41C-07 | 3.227439 | 3.011276 | 2.969992 |
| TCGA-CC-5259-01A-31R-A213-07 | 3.098416 | 2.803034 | 2.874295 |
| TCGA-EP-A2KA-01A-11R-A180-07 | 3.085249 | 2.767742 | 2.965629 |
| TCGA-DD-A1EC-01A-21R-A131-07 | 3.112268 | 2.746038 | 2.971845 |
| TCGA-DD-AAD3-01A-11R-A41C-07 | 3.208297 | 2.986296 | 3.035914 |
| TCGA-DD-AACS-01A-11R-A41C-07 | 3.156944 | 2.919476 | 2.980583 |
| TCGA-BD-A3ER-01A-11R-A213-07 | 3.193162 | 2.966045 | 3.025419 |
| TCGA-GJ-A6C0-01A-12R-A311-07 | 3.1767 | 2.956059 | 3.094037 |
| TCGA-2Y-A9GS-01A-12R-A38B-07 | 3.146056 | 2.986628 | 3.041012 |
| TCGA-BC-A8YO-01A-11R-A37K-07 | 3.102813 | 2.845734 | 3.0777 |
| TCGA-CC-A9FW-01A-11R-A37K-07 | 3.200856 | 2.791021 | 3.020723 |
| TCGA-DD-A1ED-01A-11R-A155-07 | 3.243352 | 2.97056 | 2.964294 |
| TCGA-EP-A12J-01A-11R-A131-07 | 3.167333 | 2.872486 | 2.944348 |
| TCGA-DD-A1EJ-01A-11R-A155-07 | 3.156651 | 2.816565 | 3.064241 |
| TCGA-G3-AAV2-01A-11R-A37K-07 | 3.164455 | 2.853203 | 2.901048 |
| TCGA-DD-AADY-01A-11R-A41C-07 | 3.115366 | 2.767294 | 2.981697 |
| TCGA-CC-5263-01A-01R-A131-07 | 2.992364 | 2.747806 | 3.062616 |
| TCGA-DD-AADP-01A-11R-A39D-07 | 3.251993 | 2.949792 | 2.984687 |
| TCGA-BC-A69I-01A-11R-A311-07 | 3.223485 | 2.902023 | 2.922348 |
| TCGA-G3-AAV1-01A-11R-A38B-07 | 3.196421 | 2.883386 | 3.017257 |
| TCGA-O8-A75V-01A-11R-A32O-07 | 3.178977 | 2.959277 | 2.965187 |
| TCGA-ZP-A9CY-01A-11R-A38B-07 | 3.233959 | 3.033837 | 2.995611 |
| TCGA-G3-A7M8-01A-11R-A33R-07 | 3.221074 | 2.930358 | 2.98924 |
| TCGA-DD-AAE0-01A-11R-A41C-07 | 3.026571 | 2.642319 | 2.936717 |
| TCGA-ZS-A9CE-01A-11R-A37K-07 | 3.192732 | 2.926118 | 2.966916 |
| TCGA-DD-A73B-01A-12R-A32O-07 | 3.215828 | 2.876966 | 3.055681 |
| TCGA-RC-A7SB-01A-21R-A352-07 | 3.217971 | 2.963034 | 2.979659 |
| TCGA-DD-AADN-01A-11R-A41C-07 | 3.108706 | 2.707822 | 2.972746 |
| TCGA-CC-A5UE-01A-11R-A28V-07 | 3.126858 | 2.84071 | 2.94169 |
| TCGA-DD-AACZ-01A-11R-A41C-07 | 3.121515 | 2.67803 | 3.056295 |
| TCGA-DD-AAVQ-01A-11R-A41C-07 | 3.109735 | 2.832099 | 2.936242 |
| TCGA-BD-A3EP-01A-11R-A22L-07 | 3.137994 | 2.895512 | 3.001062 |
| TCGA-DD-A39V-01A-11R-A213-07 | 3.14463 | 2.801848 | 2.937931 |
| TCGA-NI-A4U2-01A-11R-A28V-07 | 3.207242 | 2.900543 | 2.944025 |
| TCGA-DD-A4NF-01A-11R-A27V-07 | 3.175983 | 2.848353 | 2.93963 |
| TCGA-DD-A11A-01A-11R-A131-07 | 3.261539 | 2.941754 | 2.987807 |
| TCGA-DD-AAD0-01A-11R-A41C-07 | 3.111403 | 2.779681 | 3.065751 |
| TCGA-DD-A3A6-01A-11R-A22L-07 | 2.862645 | 2.537029 | 2.82322 |
| TCGA-DD-A114-01A-11R-A131-07 | 3.113736 | 2.84973 | 3.047248 |
| TCGA-5C-AAPD-01A-21R-A39D-07 | 3.129446 | 2.819293 | 2.983339 |
| TCGA-HP-A5N0-01A-11R-A28V-07 | 3.174725 | 2.920972 | 2.987985 |
| TCGA-T1-A6J8-01A-11R-A32O-07 | 3.196812 | 2.984347 | 3.020289 |
| TCGA-XR-A8TC-01A-11R-A36F-07 | 3.208333 | 2.825004 | 2.980773 |
| TCGA-DD-AACK-01A-11R-A41C-07 | 3.16542 | 2.894097 | 3.040984 |
| TCGA-MI-A75C-01A-11R-A32O-07 | 3.160996 | 2.891213 | 2.958571 |
| TCGA-DD-AACP-01A-11R-A41C-07 | 3.180975 | 2.820026 | 3.096864 |
| TCGA-DD-AACT-01A-11R-A41C-07 | 3.093179 | 2.861329 | 2.991096 |
| TCGA-DD-A73E-01A-12R-A32O-07 | 3.107775 | 2.842509 | 2.960113 |
| TCGA-BC-4072-01B-11R-A155-07 | 3.148708 | 2.821096 | 2.972754 |
| TCGA-BC-4073-01B-02R-A131-07 | 3.073273 | 2.830378 | 3.042675 |
| TCGA-XR-A8TG-01A-11R-A36F-07 | 3.184516 | 2.951053 | 2.959694 |
| TCGA-RC-A6M6-01A-11R-A32O-07 | 3.128241 | 2.755946 | 3.038084 |
| TCGA-BC-A69H-01A-11R-A311-07 | 3.104052 | 2.722533 | 3.010658 |
| TCGA-DD-AAC8-01A-11R-A41C-07 | 3.057058 | 2.672546 | 2.943093 |
| TCGA-DD-AADJ-01A-11R-A41C-07 | 3.189219 | 2.859017 | 2.95347 |
| TCGA-2Y-A9H9-01A-21R-A39D-07 | 3.268188 | 2.909692 | 2.974389 |
| TCGA-ED-A5KG-01A-11R-A27V-07 | 3.014408 | 2.619446 | 2.991414 |
| TCGA-DD-AACO-01A-11R-A41C-07 | 3.185693 | 2.885311 | 2.859107 |
| TCGA-G3-A25Z-01A-11R-A16W-07 | 3.216196 | 2.868198 | 2.98567 |
| TCGA-DD-A4NV-01A-11R-A311-07 | 3.228808 | 3.001743 | 3.020498 |
| TCGA-FV-A2QR-01A-11R-A213-07 | 3.143827 | 2.860467 | 3.020308 |
| TCGA-G3-AAV7-01A-11R-A38B-07 | 3.045814 | 2.743596 | 3.025195 |
| TCGA-2Y-A9H4-01A-11R-A38B-07 | 3.236935 | 2.913992 | 2.954218 |
| TCGA-3K-AAZ8-01A-12R-A39D-07 | 3.230277 | 2.86626 | 2.96375 |
| TCGA-CC-5261-01A-01R-A131-07 | 3.065922 | 2.800473 | 2.964219 |
| TCGA-ZP-A9D2-01A-11R-A38B-07 | 3.069885 | 2.740516 | 2.95713 |
| TCGA-2Y-A9H5-01A-11R-A38B-07 | 3.138312 | 2.952891 | 2.948512 |
| TCGA-G3-A25T-01A-11R-A16W-07 | 3.02068 | 2.782633 | 2.920308 |
| TCGA-DD-AAEA-01A-11R-A41C-07 | 3.201717 | 2.883855 | 2.996944 |
| TCGA-RG-A7D4-01A-12R-A33R-07 | 3.115478 | 2.761937 | 2.974616 |
| TCGA-DD-AACD-01A-11R-A41C-07 | 3.205961 | 2.883808 | 2.925787 |
| TCGA-DD-AAD8-01A-11R-A41C-07 | 3.160405 | 2.864537 | 2.986145 |
| TCGA-5R-AAAM-01A-12R-A41C-07 | 3.203349 | 3.048468 | 3.025509 |
| TCGA-EP-A3JL-01A-11R-A213-07 | 3.213671 | 2.968278 | 2.945679 |
| TCGA-DD-A73G-01A-22R-A32O-07 | 3.198249 | 2.793092 | 2.908816 |
| TCGA-G3-AAV4-01A-11R-A38B-07 | 3.077089 | 2.811477 | 2.974303 |
| TCGA-ED-A459-01A-11R-A266-07 | 3.181729 | 2.908389 | 3.028611 |
| TCGA-DD-A73C-01A-12R-A33J-07 | 3.14542 | 2.922373 | 3.053976 |
| TCGA-MR-A520-01A-11R-A266-07 | 3.226994 | 3.022298 | 2.994048 |
| TCGA-G3-A7M5-01A-11R-A33R-07 | 3.225724 | 2.90435 | 2.983051 |
| TCGA-MI-A75G-01A-11R-A32O-07 | 3.23291 | 2.906449 | 2.929894 |
| TCGA-CC-A7IK-01A-12R-A33R-07 | 3.125308 | 2.730632 | 2.967093 |
| TCGA-G3-A5SJ-01A-11R-A27V-07 | 3.10342 | 2.812925 | 2.977743 |

**Reference**

1. Hamabe A, Konno M, Tanuma N, Shima H, Tsunekuni K, Kawamoto K, Nishida N, Koseki J, Mimori K, Gotoh N, et al: **Role of pyruvate kinase M2 in transcriptional regulation leading to epithelial–mesenchymal transition.** 2014, **111:**15526-15531.
